# Dysregulated anti-oxidant signalling and compromised mitochondrial integrity negatively influence regulatory T cell function and viability in liver disease

**DOI:** 10.1016/j.ebiom.2023.104778

**Published:** 2023-08-30

**Authors:** Trishan Vaikunthanathan, Emmanuelle Landmann, Diana Marin Correa, Marco Romano, Silvia Cellone Trevelin, Qi Peng, Elena Crespo, Mauro Corrado, Juan-José Lozano, Erika L. Pearce, Elena Perpinan, Anna Zoccarato, Leonard Siew, Joy Edwards-Hicks, Reenam Khan, Nguyet-Thin Luu, Mark R. Thursz, Philip N. Newsome, Marc Martinez-Llordella, Naina Shah, Robert I. Lechler, Ajay M. Shah, Alberto Sanchez-Fueyo, Giovanna Lombardi, Niloufar Safinia

**Affiliations:** a Department of Inflammation Biology, Institute of Liver Studies, School of Immunology and Microbial Sciences, James Black Centre, King's College London, London, SE5 9NU, United Kingdom; b Peter Gorer Department of Immunobiology, School of Immunology and Microbial Sciences, King's College London, 5th Floor, Tower Wing, Guy's Hospital, Great Maze Pond, London, SE1 9RT, United Kingdom; c Bloomberg-Kimmel Institute for Cancer Immunotherapy and Department of Oncology, Johns Hopkins University School of Medicine, Baltimore, MD, USA; d Cologne Excellence Cluster on Cellular Stress Responses in Aging-Associated Diseases (CECAD), Joseph Stelzmannstrasse 26, 50931, Cologne, Germany; e Centro de Investigación Biomédica en Red Enfermedades Hepáticas y Digestivas (CIBEREHD), Calle Rossello 153 Bajos, O8036, Barcelona, Spain; f Department of Immunometabolism, Max Planck Institute of Immunobiology & Epigenetics, Stübeweg 51, 79108, Freiburg, Germany; g Centre for Liver and Gastroenterology Research and Birmingham National Institute for Health Research (NIHR) Birmingham Biomedical Research Centre, Institute of Immunology and Immunotherapy, University of Birmingham, Birmingham, United Kingdom; h Division of Digestive Diseases, Department of Metabolism, Digestion and Reproduction, Imperial College London, Liver Unit, 10th Floor QEQM Building, St Mary’s Hospital, W2 1NY, London, United Kingdom; i Institute of Liver Sciences, King's College Hospital NHS Foundation Trust, London, SE5 9NU, United Kingdom; j James Black Centre, Department of Cardiovascular sciences, British Heart Foundation Centre of Excellence, School of Cardiovascular and Metabolic Medicine and Sciences, King's College London, London, SE5 9NU, United Kingdom

**Keywords:** Liver cirrhosis, Regulatory T cells, Redox homeostasis, Nrf2/heme oxygenase-1, Mitochondria, Oxidative stress

## Abstract

**Background:**

Dysregulated inflammatory responses and oxidative stress are key pathogenic drivers of chronic inflammatory diseases such as liver cirrhosis (LC). Regulatory T cells (Tregs) are essential to prevent excessive immune activation and maintain tissue homeostasis. While inflammatory cues are well known to modulate the function and stability of Tregs, the extent to which Tregs are influenced by oxidative stress has not been fully explored.

**Methods:**

The phenotypic and functional properties of CD4^+^CD25^+^CD127^lo/-^ Tregs isolated from patients with LC were compared to healthy controls (HC). Treg redox state was investigated by characterizing intracellular reactive oxygen species (ROS), NADPH oxidase-2 (Nox2) activity, mitochondrial function, morphology, and nuclear factor-erythroid 2-related factor (Nrf2) antioxidant signalling. The relevance of Nrf2 and its downstream target, Heme-oxygenase-1 (HO-1), in Treg function, stability, and survival, was further assessed using mouse models and CRISPR/Cas9-mediated HO-1 knock-out.

**Findings:**

Circulating Tregs from LC patients displayed a reduced suppressive function, correlating with liver disease severity, associated with phenotypic abnormalities and increased apoptosis. Mechanistically, this was linked to a dysregulated Nrf2 signalling with resultant lower levels of HO-1, enhanced Nox2 activation, and impaired mitochondrial respiration and integrity. The functional deficit in LC Tregs could be partially recapitulated by culturing control Tregs in patient sera.

**Interpretation:**

Our findings reveal that Tregs rely on functional redox homeostasis for their function, stability, and survival. Targeting Treg specific anti-oxidant pathways may have therapeutic potential to reverse the Treg impairment in conditions of oxidative damage such as advanced liver disease.

**Funding:**

This study was funded by the 10.13039/100010269Wellcome Trust (211113/A/18/Z).

**Research in context**

**Evidence before this study**

Chronic liver damage driven by both inflammation and oxidative stress often leads to cirrhosis (LC). Without treatment, patients with LC inexorably progress to clinical decompensations and death. The most advanced forms of LC are characterized by the development of systemic inflammation and innate immune deficits, known as cirrhosis-associated immune dysfunction (CAID), which is believed to contribute to multi-organ failure and death. Regulatory T cells (Tregs) are a subset of immune cells that are critical to curb excessive immune activation, to maintain immune and tissue homeostasis, and to prevent autoimmunity. The role of Tregs in the natural history of chronic liver disease as well as their involvement in the immunological abnormalities associated with LC has not been investigated in humans.

**Added value of this study**

Our study has revealed a numerical and functional deficit of circulating Tregs in patients with LC, which correlates with liver disease severity and resembles what has been described in many autoimmune diseases. In contrast to most published studies describing CAID, our analyses were conducted on specimens collected from stable LC patients without acute decompensations, thus avoiding the risks of clinical confounders (e.g. sepsis, extra-hepatic organ failure, medications). From a mechanistic standpoint, we have identified a dysregulated intracellular redox homeostasis as a key element for the Treg abnormalities observed in LC, which is mainly driven by the impairment of the anti-oxidant Nrf2 signalling pathway and a resultant loss of mitochondrial function and integrity. Furthermore, our findings demonstrate the role of the cytoprotective enzyme HO-1, a downstream target of Nrf2 signalling, in promoting Treg survival under oxidative stress conditions.

**Implications of all the available evidence**

Our finding that in LC patients at early stages of their disease circulating Tregs are already dysfunctional fills a gap in our understanding of the immunopathogenesis of the disease. What our results suggest is that clinically stable LC are already predisposed to develop the dysregulated inflammatory responses that constitute the hallmark of decompensated LC and that contribute to poor prognosis. This implies that it may be possible to prevent clinical decompensations and LC progression, thus improving patient survival, by therapeutically restoring Treg function. In addition, from our results demonstrating the importance of redox homeostasis in the maintenance of Treg viability and function, we infer that similar mechanisms could be at play in other pathological conditions mediated by oxidative damage that also influence Treg function (e.g. ageing, cancer, cardiovascular diseases). The availability of multiple druggable targets in the redox homeostasis machinery provide potential opportunities to correct Treg dysfunction in these settings. A note of caution is however due, given the need to conduct further studies to identify the specific mediators responsible for Treg dysfunction in LC, and to establish links between the magnitude of the dysfunction and clinical outcomes.

## Introduction

CD4^+^CD25^+^Foxp3^+^ Regulatory T cells (Treg) are known to modulate inflammatory responses by suppressing effector T cells, inhibiting neutrophils and pro-inflammatory macrophages, and promoting the activities of anti-inflammatory macrophages and monocyte subsets.^1^ Tregs are of paramount importance to maintain immune homeostasis, as their dysfunction leads to the development of systemic inflammation, autoimmunity, and immunopathology.^2^ Under steady state, Tregs maintain a stable immunosuppressive phenotype. However, in certain inflammatory environments (e.g. IL-6,^3^^,^^4^ TNFα^5^^,^^6^) Treg function can be compromised in number and/or function,^7^, ^8^, ^9^ exacerbating the underlying disease. Moreover, our own previous work indicates that reactive oxygen species (ROS), inherently associated to inflammation as a result of immune cell activation,^10^^,^^11^ can also induce Treg dysfunction.^12^^,^^13^ Thus, we described a link between the expression of the ROS-generating NADPH oxidase-2 (Nox2) enzyme and Treg phenotype and function, with Nox2-deficient Tregs exhibiting higher suppressive capacity than wild type Tregs, both in vitro and in vivo.^12^^,^^13^

Among the various signalling pathways modulating intracellular redox balance, the transcription factor nuclear factor-erythroid 2-related factor (Nrf2) serves as a primary cellular defence against the cytotoxic effects of oxidative stress.^14^ In response to inflammation and ROS, Nrf2 translocates to the nucleus^14^^,^^15^ and upregulates a variety of antioxidant, anti-apoptotic and anti-inflammatory enzymes, among others heme-oxygenase-1 (HO-1).^14^^,^^16^^,^^17^ HO-1 exerts powerful cytoprotective effects in various chronic inflammatory diseases^18^ through the degradation of heme, a pro-oxidant molecule whose bioavailability is increased as a result of tissue injury, into its by-products ferrous iron, carbon monoxide and biliverdin.^19^^,^^20^ In addition to its anti-inflammatory properties, several studies have recently highlighted a role for HO-1 in protecting cells from oxidative-mediated damage by regulating mitochondrial function with activation of the Nrf2/HO-1 pathway as a mechanism to overcome ROS challenge.^21^, ^22^, ^23^ HO-1 is constitutively expressed by human Tregs^24^ and is involved in FOXP3-mediated suppression,^25^ in the production of IL-10^26^ and in the protection of Tregs from cellular damage.^27^ Taken together with our previous findings,^13^ the evidence outlined above raise the possibility, yet to be investigated, of the Nrf2/HO-1 and Nox2 balance being a key determinant of Treg function in clinical settings characterized by both excessive inflammation and oxidative stress.

Liver cirrhosis (LC) constitutes the end stage of most chronic inflammatory liver diseases. Left unchecked, LC evolves towards the development of acute clinical decompensations, multi-organ failures, and eventually death.^28^ As shown in a multiplicity of studies, LC is associated with excessive oxidative stress^29^, ^30^, ^31^, ^32^ and leads to a severely dysregulated systemic immune system, characterised by both immunodeficiency and increased inflammation.^33^^,^^34^ This phenomenon is believed to be responsible for the propensity of LC patients to develop extra-hepatic organ failures, as well as for their increased susceptibility to infections and their high mortality as a result of sepsis.

In the current study, we sought to investigate the extent to which intracellular redox balance influences human Treg homeostasis and function, using LC as paradigmatic disease model of oxidative stress and systemic inflammation. To this end, we conducted an exhaustive phenotypic and functional characterisation of circulating Tregs from clinically stable LC patients. Furthermore, we performed mechanistic investigations to elucidate whether the expression and activity of anti-oxidant and pro-oxidant enzymes alters Treg function using both clinical specimens and a relevant animal model. This study sheds new light on the impact of excessive oxidative stress on Treg function in humans. We propose that modulation of Treg redox homeostasis may represent a strategy to counteract the dysregulated systemic inflammation driving clinical complications in LC patients.

## Methods

### Study participants

A total of 108 patients with stable LC were prospectively recruited from the liver outpatient clinic at King's College Hospital from September 2015 until June 2023. From these patients, 103 were diagnosed with alcohol-related cirrhosis (ARC), abstinent for at least 6 months, and 5 patients with non-alcoholic steatohepatitis (NASH). In parallel 71 healthy controls (HC) were recruited and used as comparators (Table 1).

**Table 1:** Demographic and clinical characteristics of study participants.

	HC (n = 71)	LC (ARC, n = 103)	LC (NASH, n = 5)
Age, years	49.9 (10.9)	57.6 (10.9)	57.8 (4.8)
Sex, number of males (%)	44 (62%)	77 (75%)	3 (60%)
MELD Score		12 (8–14)	13 (13–14)
Haemoglobin, g/L		131.5 (121.8–142.3)	132 (110–137)
Platelet count, x10^9^/L		113 (87–175.8)	108 (100–124)
INR		1.2 (1.1–1.4)	1.4 (1.4–1.5)
Bilirubin, μmol/l		28 (13–36.3)	36 (29–38)
AST, IU/ml		34 (24–48.8)	38 (33–49)
ALP, IU/ml		113.5 (89.5–152)	120 (110–147)
GGT, IU/l		85 (41.8–151)	73 (60–84)
Albumin, g/L		39 (34–45.3)	31 (29–32)
Creatinine, μmol/l		74 (62.8–95.3)	80 (65–97)

All data is presented as median (IQR), except for age, which is shown as mean (SD).

Healthy control (HC), Liver cirrhosis (LC), Alcohol-related cirrhosis (ARC), Non-alcoholic liver steatohepatitis (NASH); Standard deviation (SD), Model of end stage liver disease score (MELD), Aspartate Transaminase (AST); Alkaline Phosphatase (ALP), Gamma-glutamyl Transferase (GGT).

To eliminate confounders known to influence lymphocyte function, patients with infections in the preceding three months, acute-on-chronic liver failure, previous transplantation, autoimmune diseases, hepatotropic viral disease, cholestatic liver disease, haemochromatosis, hepatocellular carcinoma, and/or those on iron supplementation or antibiotics (including rifaximin) were excluded.

### Ethics and study approval

The study was conducted in line with the Declaration of Helsinki and approved by the Institutional Review Board of Guy's hospital (reference 09/H0707/86). Informed consent was obtained from all subjects enrolled into the study. All blood samples were handled and disposed of in accordance with the UK Human Tissue Act 2008. The animal study was performed in accordance with all legal, ethical and institutional requirements and was approved by the UK Home office (PPL 7009066).

### Treg isolation

Peripheral blood mononuclear cells (PBMC) were isolated from whole blood by Ficoll density gradient centrifugation. CD4^+^CD25^+^ T cells were isolated by negative selection of CD4^+^ T cells followed by positive selection of CD25^+^ T cells using the human CD4^+^CD25^+^ T Regulatory Cell Isolation Kit (130-091-301, Miltenyi Biotec). The purity of the isolated cell product was between 90 and 98%. Aliquots of the HC CD4^+^CD25^-^ effector T cells (Teff) were cryopreserved and used as responder cells in subsequent suppression assays, as described below.

For some experiments CD4^+^CD25^+^CD127^lo/-^ Tregs were isolated using flow cytometry-based sorting. In brief, CD4^+^ T cells were enriched by negative selection from PBMCs, as described above, and subsequently stained with anti-CD4, anti-CD25 and anti-CD127 antibodies. Tregs were sorted based on CD4^+^CD25^+^CD127^lo/-^ on a three laser FACS Aria high speed cell sorter (BD Biosciences).

To isolate healthy CD4^+^CD25^+^ Tregs for culture in HC or LC serum, PBMCs from healthy donors was obtained from anonymized human leukocytes cones, supplied by the National Blood Transfusion Service (NHS Blood and Transplantation, Tooting, London, UK). CD4^+^ T cells were enriched using Rosette-Sep Human CD4^+^ T cell enrichment cocktail (15062, Stem Cell Technologies) according to the manufacturer's protocol and isolated by Ficoll density gradient centrifugation. CD25^+^ T cells were then labelled and separated from CD4+ T cells by positive magnetic separation using Human CD25 Microbeads II and LS columns (130-092-983, Miltenyi Biotec).

### Flow cytometry phenotyping

Flow cytometry samples were acquired on a BD LSRFortessa flow cytometer (BD Biosciences) and analysed using the latest FlowJo software. For the staining, we first labelled dead cells with the Live/Dead Fixable dead cell stain kit (Violet L34964, Green L34970, Near-IR Red L34975; Invitrogen) in Phosphate-Buffered Saline (PBS, Gibco) for 10 min on ice. Staining of extracellular markers was performed in Staining Buffer (PBS + 0.9% Bovine Serum Albumin (BSA; Sigma Aldrich)) for 30 min on ice. In case of intracellular antigen labelling, the cells were fixed and permeabilized using the eBioscience Fixation/Permeabilization reagents (Fixation/Permeabilization Concentrate 00-5123-43, Fixation/Permeabilization Diluent 00-5223-56; ThermoFisher) for 30min at RT.

To label apoptotic human Tregs, cells were stained with Annexin V-FITC (640906, Biolegend) in Annexin V binding buffer (422201, Biolegend) for 15min on ice. To quantify apoptosis of murine Tregs, enriched WT or Nrf2^−/−^ Tregs were stimulated using plate-bound anti-CD3 (2 μg/ml, BD Biosciences) and anti-CD28 (4 μg/ml, R&D systems) for 72 h and stained for Annexin V–FITC and propidium iodide using a Annexin V Apoptosis Detection kit (640914; Biolegend) as per manufacturer's protocol.

ROS was investigated by incubating the cells with 10 μM dihydroethidium (DHE; D11347, Invitrogen;^35^) for 30 min at 37 °C followed by a washing step in Hanks’ balanced salt solution (HBSS) without phenol red supplemented with diethylenetriamine-pentacetic acid (DTPA, 100 μM). The fluorescent probe BODIPY 581/591 1 μg/ml (D3861, Invitrogen) was used as an indicative of lipid peroxidation by LC or HC Tregs according to previous published reports.^36^ The levels of cell surface thiol groups were determined by 15 min incubation of CD4^+^CD25^+^ cells with Alexa 633-maleimide (10 μM; A20342; Invitrogen) at 4 °C as previous described.^37^ A complete list of all commercially available fluorochrome-conjugated anti-human and anti-mouse monoclonal antibodies used in the study are summarised in Table S4.

### Culture in pro-inflammatory cytokines

To investigate the phenotypic stability of HC and LC CD4^+^CD25^+^ Tregs, cells were cultured in 2 mixes of pro-inflammatory cytokines in the presence of anti-CD3/CD28 Dynabeads (11131D, ThermoFisher) in a 1:1 cell-to-bead ratio for 5 days (37 °C, 5% CO_2_). Mix 1 included IL-2 (10 U/ml; 202-IL), IL-1β (10 ng/ml; 201-LB), IL-6 (4 ng/ml, 206-IL), TGF-β (5 ng/ml; 240-B), and Mix 2 IL-2 (10 U/ml; 202-IL), IL-21 (25 ng/ml; 8879-IL), IL-23 (25 ng/ml; 1290-IL), TGF-β (5 ng/ml; 240-B). All cytokines purchased from R&D Systems®. At the end of the culture period the cells were treated with Phorbol myristate acetate (PMA), the calcium ionophore Ionomycin and GolgiStop for 4 h at 37 °C followed by the intracellular staining for IL-17 and IFNγ.

### Cytokine bead array

To measure the cytokine concentration in HC and LC patient serum we employed the LEGENDplex Multi-Analyte Flow Assay kits (Human Cytokine panel 2 and Human Th Cytokine panel) according to the manufacturer's instructions.

### TSDR analysis

Genomic DNA of freshly isolated CD4^+^CD25^+^CD127^lo/-^ Tregs of HC and LC patients was extracted using the ZymoResearch Direct-zol DNA/RNA MiniPrep (R2080, ZymoResearch) kit according to the manufacturer's protocol and analysed for TSDR methylation (FOXP3 Methylation Panel ID N70V3P14) by EpiGenDx. In short, the DNA was bisulfite converted using the EZ-96 DNA Methylation Direct Kit (ZymoResearch). PCR of bisulfite modified DNA was performed using the HotStarTaq Polymerase (Qiagen). Following a barcoding and enrichment step on the Ion ChefTM system using Ion 520TM & Ion 530TM ExT Chef reagents, the samples were subject to sequencing by the Ion S5TM sequencer using an Ion 530TM sequencing chip. Result files were aligned to a local reference database using the open-source Bismark Bisulfite Read Mapper program (v0.12.2) with the Bowtie2 alignment algorithm (v2.2.3). The Methylation levels were calculated as % methylation = (number of methylated reads/total number of reads).

### Suppression assays

Cryopreserved third-party HC CD4^+^CD25^-^ Teffs were thawed and labelled with 5 mM CFSE (Cell Division tracker kit 423801, Biolegend). 1 × 10^5^ stained Teff were co-cultured alone or together with CD4^+^CD25^+^CD127^lo/-^ Tregs of HC or LC at different Tregs to Teff ratio (1:1, 1:5 and 1:10) in X-VIVO 15 medium (BE02-053Q, Lonza) supplemented with 5% human heat-inactivated AB serum (Merck Life Science) and activated with anti-CD3/CD28-coated Dynabeads (11131D, Gibco Thermofisher) in a bead-to-cell ratio 1:40. After the 5-day incubation period at 37 °C and 5% CO_2_ proliferation of Teffs was determined as dilution of the CFSE dye. The suppressive ability of Tregs was assessed as the relative proliferation of Teffs in the presence of Tregs in comparison to the proliferation of Teffs alone.

### Co-localization of the NADPH-oxidase 2 (Nox2) subunits gp91^phox^ and p47^phox^ by immunofluorescence microscopy

Freshly isolated Tregs were fixed with 4% paraformaldehyde for 30 min and permeabilized in 0.2% Triton-X for further 30 min. Non-specific interactions were blocked by incubation with 2% BSA plus 1:50 horse serum solution for 30 min. Cells were incubated 12 h with the primary antibodies goat anti-gp91phox (1:200; Sc-5827, Santa Cruz Biotechnology; RRID: AB_647636) and rabbit anti-phospho-p47phox (pSer^345^) (1:100; SAB4504721, Sigma–Aldrich) and then incubated 2 h with the secondary antibodies red fluorescent Alexa Fluor 594 (donkey anti-goat; 1:400; A-11058 Invitrogen; RRID:AB_2534105), and/or green fluorescent Alexa Fluor 488 (donkey anti-rabbit; 1:200; Invitrogen; RRID:AB_2762833). Nuclei were stained with 4, 6-diamidino-2-phenylindole (DAPI; D9542; Sigma–Aldrich). Images of stained cells were captured using a confocal microscope SP5 (Leica, USA) and analysed in ImageJ (National Institutes of Health, NIH) using the plugin co-localization analysis/co-localization highlighter (co-localized points—8 bit), That tool generated a new image that presented the points of co-localization of p47phox and gp91phox, those mean of fluorescence intensities were determined in two to four cells/randomly selected field.

### Metabolic phenotyping using seahorse

Real time bioenergetics analysis of oxygen consumption rate (OCR) and extracellular acidification rate (ECAR) of freshly isolated HC and LC CD4^+^CD25^+^ Tregs was performed using the Seahorse XF24 Extracellular Flux Analyzer (Agilent Technologies). In brief, Tregs were cultured overnight in X-VIVO supplemented with 5% human AB serum and IL-2 50 U/ml and stimulated with anti-CD3/CD28-coated Dynabeads (1:5 bead-to-cell ratio; 11131D, Gibco Thermofisher) at 37 °C, 5% CO_2_. The following day cells were debeaded, washed and incubated in a 24-well Seahorse plate pre-coated with 0.01% poly-d-lysine (A3890401, Gibco) in serum free non-buffered XF media (DMEM without phenol red (D5030-10X1L; Sigma–Aldrich) containing 10 mM glucose (Sigma Aldrich), 2 mM l-glutamine (G7513, Sigma Aldrich), 1 mM sodium pyruvate (11360070 Gibco) at pH 7.4 for 1 h at 37 °C without CO_2_. OCR and ECAR real-time measurements were performed under basal conditions and after the addition of the following pharmacological inhibitors of oxidative phosphorylation: oligomycin (1 μM), carbonylcyanide-4-(trifluoromethoxy)-phenylhydrazone (FCCP, 1.5 μM), rotenone (1 μM) and antimycin A (1 μM) (all from Sigma–Aldrich). Basal OCR, maximal respiratory capacity, ATP production respiration, basal ECAR, glycolytic capacity was calculated according to the manufacturers’ protocol following normalization to cell numbers (Agilent Technologies).

### Electron microscopy imaging

For mitochondrial imaging, 0.5 × 10^6^ Treg cells were fixed in 2.5% glutaraldehyde in 100 mM sodium cocodylate, washed in cocodylate buffer. After dehydration samples were embedded in Eponate 12 resin (Ted Pella) and sections were cut. Images were acquired using a FEI Tecnai 12 Transmission electron microscope equipped with a TIETZ digital camera. Cristae width was measured using ImageJ software and averaged over 50 independent images, acquisition of EM micrographs and measurements of max cristae width displayed were performed using ImageJ software (NIH). Brightness and contrast were adjusted in ImageJ software (NIH).

### RNA sequencing

RNA was extracted from freshly isolated CD4^+^CD25^+^CD127^lo/-^ Tregs of HC and LC using the ZymoResearch Direct-zol DNA/RNA MiniPrep (R2080, ZymoResearch) kit according to the manufacturer's protocol and Standard RNA-Sequencing was performed by Genewiz (Azenta Life Sciences). Raw reads were analysed for data quality using FastQC v0.11.5 (Babraham Bioinformatics) and filtered using skewer v0.2.2^38^ for removing the low-quality reads and trimming the Illumina adapter. STAR program^39^ against Human genome (GRCh38) was used for mapping the reads followed by the quantification of genes with the RSEM program^40^ using GENCODE v26 reference annotation.^41^ After eliminating genes without an expected value greater than 10, we carried out DESeq2,^42^ a method for differential analysis of count data, using shrinkage estimation for dispersions and fold changes to improve stability. For functional pathway analyses, we applied both Gene Set Enrichment Analysis (GSEA) from the clusterProfiler R package and Single-Sample Gene Set Enrichment Analysis (ssGSEA) from the GSVA R package,^43^ using the curated gene set collection of canonical pathways (C2) and Gene Ontology (C5) available from the Molecular Signatures Database (MSigDB). Gene sets with <15 or >500 genes were excluded from the analysis. Among the top 15 pathways most significantly over- or under-represented when comparing LC and HC (Table S2), those associated with immunometabolism and redox homeostasis are shown (GSEA enrichment plots) in Figs. 2j, k and 3f.

**Fig. 1:**
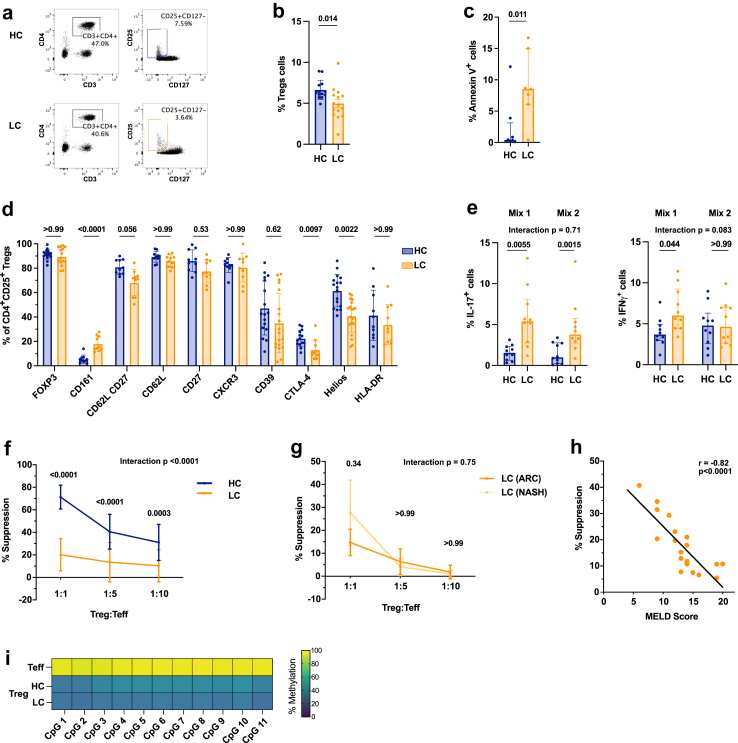
**Circulating Tregs from LC patients are reduced in number and show phenotypic instability and functional impairment. a.** Representative flow cytometry plots and **b.** summary (%) of CD3^+^CD4^+^CD25^+^CD127^lo/-^ Tregs in PBMCs of healthy controls (HC) and stable liver cirrhosis (LC) patients. HC n = 13, LC n = 15; unpaired t-test. Mean (SD). **c.** Proportion of Annexin V^+^ Tregs in HC and LC patients. HC n = 8, LC n = 7; unpaired t-test following log10 transformation. Median (IQR). **d.** Proportion (%) of CD4^+^CD25^+^CD127^lo/-^ Tregs in HC and LC PBMCs expressing different phenotypic markers. HC n = 10–17, LC n = 10–19; multiple unpaired t-tests with Bonferroni adjustment following log10 transformation. Median (IQR). **e.** Percentage of IL-17^+^FoxP3^+^ Tregs (left) and IFNγ^+^FoxP3^+^ Tregs (right) of HC and LC following a 5-day culture in pro-inflammatory cytokines (Mix 1: IL-2, IL-1β, IL-6, TGF-β. Mix 2: IL-2, IL-21, IL-23, TGF-β). HC/LC n = 10; Two-way repeated measures ANOVA with Bonferroni's correction for multiple pairwise comparison tests following log10 transformation. Median (IQR). **f.** Suppressive function of freshly isolated HC and LC Tregs to inhibit third party CD4^+^CD25^-^ effector T cell (Teff) proliferation at different Treg:Teff ratios. n = 20 in each group; Two-way repeated measures ANOVA with Bonferroni's correction for multiple pairwise comparison tests. Mean (SD). **g.** Suppressive function of HC and LC Tregs of different LC aetiologies (ARC or NASH). n = 5 in each group. Two-way repeated measures ANOVA with Bonferroni's correction for multiple pairwise comparison tests following log10 transformation. Values shown as SD. **h.** Linear regression of Treg suppression (from **f**) and disease severity as calculated by MELD score in LC patients. n = 20. Pearson's correlation coefficient. **i.** TSDR Methylation of freshly isolated healthy CD4^+^CD25^-^ Teff and CD4^+^CD25^+^CD127^lo/-^ Tregs of HC and LC patients. Mean percentage of methylation shown for each of the 11 analysed CpG islands. HC n = 7, LC n = 6. Abbreviations: Healthy control (HC), liver cirrhosis (LC), peripheral blood mononuclear cells (PBMCs), Model of end stage liver disease score (MELD), Alcohol-related cirrhosis (ARC), non-alcoholic steatohepatitis (NASH).

**Fig. 2:**
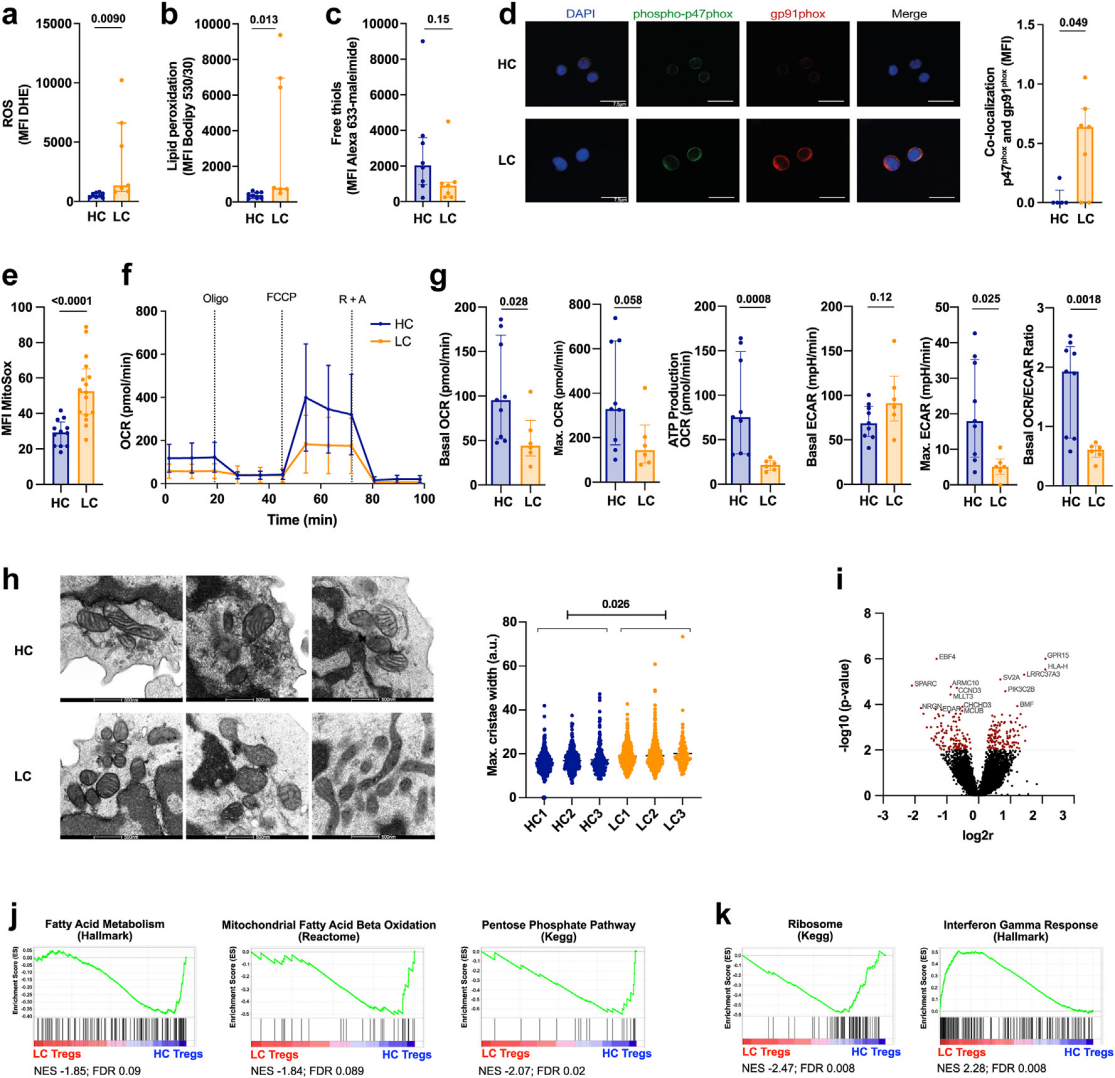
**Circulating Tregs from LC patients exhibit increased intracellular ROS and changes in mitochondria morphology. a–c.** Measurement of oxidative stress in freshly isolated HC and LC CD4^+^CD25^+^ Tregs. HC n = 8, LC n = 7; unpaired t-test with Welch's correction (a,b) following log10 transformation (a–c). Median (IQR). **a.** ROS shown by the MFI of dihydroethidium (DHE). **b.** Analysis of lipid peroxidation using the MFI of Bodipy C11 581/591. **c.** Free thiols on the cell surface indicated by the MFI of Alexa 633-maleimide. **d.** Representative confocal microscopy pictures (left) and summary (right) indicating co-localisation of the NADPH oxidase 2 (Nox2) subunits p47^phox^ (green) and gp91^phox^ (red) measured as MFI of co-localised points (white). Cell nuclei stained with DAPI (blue). Scale bar 7.5 μm. HC n = 5, LC n = 7. Mann–Whitney test. Median (IQR). **e**. Mitochondrial ROS measured by Mitosox (MFI) in HC and LC Tregs. HC n = 12, LC n = 16. Unpaired t-test following log10 transformation. Median (IQR). **f**. Metabolic profile of CD4^+^ CD25^+^ Tregs of HC and LC patients. Oxygen Consumption Rate (OCR) and extracellular acidification rate (ECAR) was measured following the exposure to Oligomycin (Oligo), FCCP and Rotenone + Antimycin A (R + A). SD. **g.** Basal and maximal OCR and ATP production (measured after Oligomycin treatment) in HC and LC Tregs. Basal ECAR, glycolytic capacity (max. ECAR) and basal OCR/ECAR ratio in HC and LC Tregs. HC n = 9, LC n = 6. Unpaired t-tests following log10 transformation. Median (IQR). **h.** Quantification of mitochondrial cristae width of HC and LC Tregs by electron microscopy. Scale bar 500 nm. HC/LC n = 3. Mean mitochondrial cristae width, HC; 16.65 (95% Confidence Interval 15.48–17.81) vs LC; 19.27 (95% Confidence Interval 18.08–20.45; p = 0.013), from linear mixed effect model (dependent variable, cristae width; fixed-effects variable, disease state; random effects variable, patient ID. Sensitivity analysis with log10 transformed cristae width p = 0.026). **i.** Volcano plot indicating the 15 top differentially expressed genes in LC Tregs. Genes with a p-value <0.01 were considered differentially expressed between LC and HC Tregs and marked in red. n = 7. **j.** Gene set enrichment analysis (GSEA) of fatty acid metabolism, fatty acid beta oxidation, and Pentose Phosphate Pathway underrepresented in LC Tregs. n = 7. **k.** GSEA of Ribosome and Interferon Gamma Response pathway genes in LC Tregs. n = 7. Abbreviations: Reactive oxygen species (ROS), Healthy control (HC), liver cirrhosis (LC), dihydroethidium (DHE), 4.6-diamino-2-phenylin-dole (DAPI), NADPH oxidase 2 (Nox2), Oxygen consumption rate (OCR), Extracellular acidification rate (ECAR), Oligomycin (Oligo), Carbonyl cyanide-4 (trifluoromethoxy) phenylhydrazone (FCCP), Rotenone + Antimycin A (R + A).

**Fig. 3:**
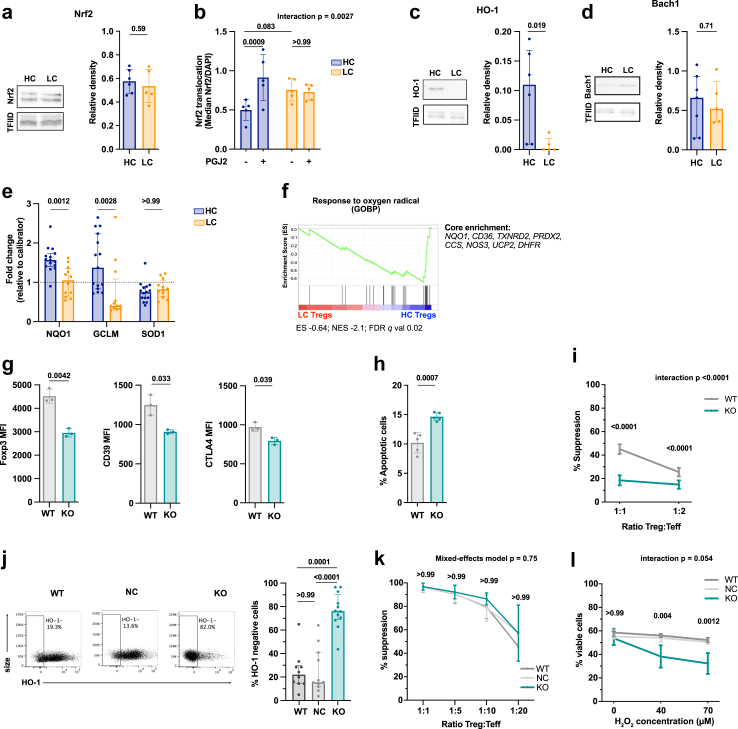
**The Nrf2/HO-1 redox pathway is dysregulated in Tregs from LC patients. a.** Nrf2 abundance in freshly isolated CD4^+^CD25^+^ Tregs of healthy controls (HC) and liver cirrhotic (LC) patients relative to the loading control TFIID measured by Western Blot. HC n = 6, LC n = 5; unpaired t test. Mean (SD). **b.** Nuclear Nrf2 translocation following 12-h prostaglandin J2 (PGJ2) stimulation in freshly isolated HC and LC Tregs measured by ImageStream. HC/LC n = 5. Two-way repeated measures ANOVA with Bonferroni's correction for multiple pairwise comparison tests. Mean (SD). **c–d.** Relative abundance of HO-1 (**c.**) and Bach1 (**d.**) in freshly isolated HC and LC Tregs relative to the loading control TFIID measured by Western Blot. HO1: HC n = 6, LC n = 5; Bach1: HC n = 7, LC n = 5; unpaired t-test following log10 transformation. Median (IQR). **e.** Relative gene expression of Nrf2 targets *NQO1*, *GCLM* and *SOD1* in freshly isolated HC and LC Tregs (normalised to a HC calibrator sample). HC n = 15, LC n = 12. Multiple unpaired t-tests with Bonferroni's adjustment following log10 transformation. Median (IQR). **f.** GSEA of oxygen radical response in HC and LC patients. Top enriched genes are listed to the right. n = 7. **g–i.** Phenotype and function of murine Nrf2^−/−^ Tregs. **g.** Expression (MFI) of phenotypic markers (FoxP3 left, CD39 middle, CTLA-4 right) in freshly isolated Tregs of WT and Nrf2^−/−^ mice. n = 3, multiple unpaired t-tests with Bonferroni's adjustment. Mean (SD). **h.** Proportion of Annexin V^+^ CD4^+^CD25^+^ Tregs from WT and Nrf2^−/−^ mice following 3-days of CD3/CD28 stimulation. n = 5, unpaired t-test. Mean (SD). **i.** Suppressive function of Nrf2^−/−^ Tregs at different Treg:Teff ratios. n = 7, Two-way repeated measures ANOVA with Bonferroni's correction for multiple pairwise comparison. Mean (SD). **j–l.** CRISPR/Cas9 mediated HO-1 knock-out in human Tregs. **j.** Representative flow cytometry plot (left) and summary (right) of CRISPR/Cas9-mediated human HO-1 knock-out Tregs. Control conditions are wild-type (WT) Tregs and negative control (NC) Tregs (i.e. electroporated with a CRISPR/Cas9 complex not targeting the human genome). WT n = 10, NC n = 11, KO n = 12. Ordinary one-way ANOVA with Bonferroni's correction for multiple pairwise comparison tests following log10 transformation. Median (IQR). **k.** Suppressive capacity of modified Tregs (KO) and its controls (WT, NC) to inhibit Teff proliferation at various Treg:Teff ratios. n = 3 (NC) n = 4 (WT, KO). Linear mixed effect model with Bonferroni's correction for multiple pairwise comparison. (SD). p-values indicate comparison between NC and KO. **l.** Percentage of viable WT, NC and HO-1 KO Tregs following 18-h incubation at different concentrations of Hydrogen Peroxide (H_2_O_2_). WT/NC/KO n = 3. Two-way repeated measures ANOVA with Bonferroni's correction for multiple pairwise comparison (SD). p-values indicate comparison between NC and KO. Abbreviations: Healthy control (HC), liver cirrhosis (LC), Nuclear factor erythroid 2-related factor 2 (Nrf2). Heme-oxygenase 1 (HO-1). NAD(P)H quinone dehydrogenase 1 (NQO1). Glutamate-cysteine ligase modifier subunit (GCLM). Superoxide dismutase 1 (SOD1). Prostaglandin J2 (PGJ2). Gene Set enrichment analysis (GSEA), Wild-type (WT), negative control (NC), knock-out (KO).

### Western Blot

Immunoblotting was performed using standard methods. Briefly, Tregs were lysed in a commercial buffer (Sigma–Aldrich, R0278) containing protease inhibitors (Sigma–Aldrich, 04693124001). The total protein in each sample was determined with Bradford reagent (Sigma–Aldrich, B6916) and 20 μg of protein was separated in 10% sodium dodecyl sulfate polyacrylamide gel electrophoresis (NuPAGE Bis-Tris gel, Life Technologies, NP0321). The proteins were transferred to nitrocellulose membranes (GE Healthcare life sciences, 10600002). The membranes were blocked with 5% milk powder dissolved in Tris-buffered saline, incubated for 12 h with anti-NRF2 (1:2000), anti-HO-1 (1:200), anti-Bach1 (1:1000) or anti-TFIID (1:1000) antibodies. Anti-rabbit (1:5000) and anti-rat (1:3000) secondary antibodies were conjugated with HRP (Sigma–Aldrich). The membranes were cut based on a standard colorimetric protein ladder before incubation with the antibodies. The signals were analysed by autoradiography after addition of luminol (Amersham ECL reagent, GE Healthcare life sciences). Optical density of the signals on the film was quantified using ImageJ software (NIH).

### Nuclear Nrf2 translocation by ImageStream

Levels of nuclear Nrf2 were evaluated in freshly isolated CD4^+^CD25^+^ Tregs (500-5′000 cells per group) following 12 h stimulation with prostaglandin J2 (PGJ2) using imaging flow cytometry. Nuclei were stained with DAPI. Cell images were acquired using an ImageStreamX MKII (Merck Millipore) and analysed with the IDEAS 6.2 software (NIH). The degree of nuclear translocation was assessed by quantifying the peak pixel intensity values for NRF2 staining within the digitally masked nuclear region (DAPI) in each individual sample.

### Preparation of cell lysates for ELISA

Snap frozen cell pellets of freshly isolated Tregs of HC and LC patients were thawed on ice. RIPA Cell Lysis Buffer 2 (Enzo Life Sciences, ADI-80-1284) containing a protease inhibitor cocktail (1:200 dilution, Sigma–Aldrich, P1860-1 ML) was added at a concentration of 4 × 10^6^ cells/ml. The cell pellet was resuspended by gentle pipetting and homogenised by passing through a 25G needle (BD Medical, 305125). The suspension was incubated on ice for 30 min and mixed intermittently. Following this the suspension was centrifuge at 21,000 g for 10 min in a 4 °C refrigerated microfuge.

### Reverse transcription quantitative real-time polymerase chain reaction (RT-qPCR)

The expression pattern of 5 Nrf2 targets genes (*NQO1, GCLM, SOD1, GPX2, GSTA1*) and 1 housekeeping gene (*ACTB*) was measured in freshly isolated peripheral blood CD4^+^CD25^+^ Tregs of HC and LC individuals by qPCR using the QuantStudio 7 Flex Real Time PCR System (Applied Biosystems). Total RNA was extracted using the Quick-RNA MiniPrep kit (R1054, ZymoResearch) according to the manufacturer's protocol and reverse transcription was performed using the Reverse transcription High Capacity cDNA Reverse Transcription Kit with RNase Inhibitor (Applied Biosystems, Cat. 4374966). qPCR reactions were run with TaqMan Universal MasterMix II, no UNG (Applied Biosystems, Cat. 4440040) and TaqMan Gene Expression Assay Probes (Applied Biosystems; *ACTB* Hs01060665_g1 (VIC), *NQO1* Hs01045993_g1 (FAM), *GCLM* Hs00978072_m1 (FAM), *SOD1* Hs00533490_m1 (FAM), GPX2 Hs01591589_m1 (FAM) GSTA2 Hs00747232_mH (FAM)). To quantify the mRNA transcript levels, the expression of target genes was normalized to the housekeeping gene *ACTB* and the results were presented as relative expression between cDNA of the target samples and a calibrator sample (independent healthy donor) according to the comparative ΔCT method. All qPCR experiments were performed in duplicates.

### Nrf2^−/−^ mice

Nrf2 knock-out (Nrf2^−/−^; RRID:IMSR_JAX:017009) and C57BL/6 wild-type (WT; RRID:IMSR_JAX:000664) mice were housed in a specific pathogen-free (SPF) condition in accordance with institutional guidelines and ethical regulations.

CD4^+^CD25^+^ Tregs were enriched from the spleen and peripheral lymph nodes (pLN) using the Dynabeads Flowcomp murine CD4^+^CD25^+^ Treg cells kit^44^ as per manufacturer's protocols (ThermoFisher Scientific). The purity of CD4^+^CD25^+^ cells was above 95%. In some cases, WT CD4^+^CD25^−^ Teff were cryopreserved for in vitro suppression assay. Similarly, antigen presenting cells (APCs) were isolated from the spleen and pLN of C57BL/6 WT mice using a magnetic negative selection protocol as described earlier.^45^ Cells were cultured in RPMI-1640 supplemented with 10% fetal calf serum, 100 U/ml penicillin, 100 μg/ml streptomycin, 2 mM l-glutamine, 10 mM HEPES and 50 μM β2-mercaptoethanol (all from ThermoFisher Scientific). In vitro suppression assays were set up by co-culturing 0.5 × 10^5^ CFSE-labelled Teff with 1 × 10^5^ WT APCs in the presence of 1 μg/ml anti-CD3_Ɛ_ antibody (ThermoFisher Scientific). Enriched WT or Nrf2^−/−^ Tregs were added to the Teff-APC co-cultures at various Treg:Teff ratios and incubated for 3 days at 37 °C. Teff proliferation was determined by CFSE dilution and Treg suppression was calculated as the relative frequency of proliferating Teff with and without Tregs.

### CRISPR-Cas9 mediated HO-1 knock-out in human Tregs

Following flow cytometry-based isolation, Tregs were cultured in a 24-well plate at a cell density of 1 × 10^6^ cells/well in X-VIVO 15 supplemented with 5% human AB serum (H4522, Sigma–Aldrich) and human CD3/CD28 Dynabeads (11131D, Thermo Fisher) in a 1:1 bead-to-cell ratio together with human IL-2 (1000 U/ml, Aldesleukin). Fresh medium and IL-2 was replenished every 2–3 days and cultures were reactivated weekly with anti-CD3/CD28 Dynabeads. On the day of transfection Cas9 Ribonucleoproteins (RNPs) were freshly complexed. Thawed Alt-R tracrRNA (1072533, IDT, 200 mM) and Alt-R crRNA (IDT, 200 mM, sequence of crRNA targeting *HMOX1*: AGGGCCTCTGACAAATCCTG; negative control crRNA #1 1072544) were mixed in a 1:1 M ratio (120 pmol each), denatured at 95 °C for 5 min and annealed at RT for 30 min to complex an 80 μM gRNA solution. To prepare the Cas9-RNP complex, 60 pmol recombinant Cas9 nuclease (1081059, IDT, 61 μM) was mixed with the gRNA (molar ratio Cas9:gRNA = 1:2) and incubated at RT for 20 min. Prior to nucleofection, pre-activated Tregs were collected, and de-beaded by placing the resuspended Tregs on a EasySep magnet. For each electroporation, 1 × 10^6^ cells were resuspended in 20 μl Lonza supplemented P3 electroporation buffer, mixed with 60 pmol RNPs and transferred to a Nucleocuvette strip (4D-Nucelofector X Kit S, Lonza). Electroporation was performed using the 4D-Nucleofector system (Lonza) with program EH-115. Immediately after transfection, 80 μl of pre-warmed X-VIVO 15 + 5% hAB media +50 U/ml IL-2 was added to the wells and incubated at 37 °C. After 20 min the cells were transferred to 24-well plates. The following day, cells were re-activated with anti-CD3/CD28 Dynabeads in a 1:1 cell-to-bead ratio and IL-2 1000 U/ml. KO efficiency was analysed by flow cytometry and sanger sequencing 72 h after electroporation. For Sanger Sequencing, genomic DNA from CRISPR/Cas9-edited and wild-type Tregs was isolated using the ZymoResearch Quick-DNA Miniprep kit according to the manufacturer's protocol. PCR of the crRNA-targeting region in the *HMOX1* gene was performed with GoTaq G2 Green Master Mix (M782B, Promega) and sequencing was carried out by Genewiz (Azenta Life Sciences).

### Serum culture experiments

Freshly isolated CD4^+^CD25^+^ Tregs of healthy anonymized donors were cultured in X-vivo 15 supplemented with 25% serum of HC or LC patients for 24 h at 37 °C, 5% CO_2_. The cultured cells were then subject to flow cytometry, in vitro suppression assays or bioenergetic analysis as described above. In the culture conditions of Seahorse experiments anti-CD3/CD28 Dynabeads were used at a cell-to-bead ratio = 5:1.

### Statistics

Statistical analysis was carried out in GraphPad Prism 9 (Version 9.5.1 GraphPad software Inc.) and IBM SPSS Statistics (version 28.0.1.1). Normally distributed data were summarised as mean and standard deviation (SD) and non-normally distributed data as median and interquartile range (IQR). Pairwise comparisons for normally distributed data were performed with unpaired t-tests, applying Welch's correction when F-tests indicated significant difference in variances. In cases in which data was not normally distributed, we log10 transformed the values to mitigate the skewness and performed a parametric unpaired t-test analysis. We performed a non-parametric Mann–Whitney test only when log10 transformation did not improve the distribution, because non-parametric tests have less power than parametric tests when sample sizes are small. To compare three or more groups that are defined by only one factor an ordinary one-way ANOVA was applied. When comparing groups with repeated measurements, we used linear mixed-effects models, to account for the relatedness between samples from the same individual. To examine whether differences between patients and controls are influenced by a given factor, we used repeated measures two-way ANOVA, or linear mixed-effects models with an interaction term between disease status and factor, following log-transformation for skewed distributions. To account for multiple comparisons, we applied Bonferroni correction and derived adjusted p-values. We calculated correlations using Pearson's correlation coefficient. A two-tailed p-value <0.05 (following Bonferroni's correction when required) was considered as statistically significant. The statistical tests performed for each experiment are indicated in the figure legends, and a per-graph summary of the statistical analyses is outlined in Table S3. The values are either shown as mean (SD) for approximately normally distributed data or median (IQR) for non-normally distributed data. For ease of interpretation, data sets that were log10 transformed are shown as median (IQR) in Figures S1 and S2 and Table S3.

### Role of funding source

The funders had no role in study design, data collection and analysis, decision to publish or preparation of the manuscript.

## Results

### Circulating Tregs from LC patients are reduced in number and show phenotypic instability and functional impairment

To investigate the phenotype of circulating Tregs in patients with clinically stable LC, we recruited 108 patients together with 71 healthy controls (HC). The majority of patients were male, and the median Model for End-stage Liver Disease (MELD) score, as measure of liver disease severity, was 12 (Table 1). The proportion of circulating CD4^+^CD25^+^CD127^lo/-^ Tregs was significantly decreased in LC patients as compared to HCs (p = 0.014; Fig. 1a and b, Fig. S1a) despite comparable numbers of CD3^+^CD4^+^ cells (p = 0.88; Fig. S1b). Of note, while HC and LC patients were not perfectly matched in what regards age and sex (Table 1), we observed no statistically significant correlation between these 2 parameters and the number of circulating CD4^+^CD25^+^CD127^lo/-^ Tregs (data not shown). The lower Treg fraction noted in LC patients was associated with a significant increase in the frequency of Annexin V^+^ cells, indicative of a higher rate of Treg apoptosis (p = 0.011; Fig. 1c). Flow cytometric analyses revealed that, in comparison to HCs, LC patients displayed a significantly lower percentage of Tregs expressing the transcription factor Helios (p = 0.0022) and CTLA-4 (p = 0.0097; Fig. 1d and Fig. S1c). No statistically significant difference was seen in the percentage of FoxP3^+^, CD39^+^ or HLA-DR^+^ cells. Although HC and LC Tregs did not significantly differ in the relative frequency of the three Treg sub-populations, as reported by Miyara et al.^46^ and characterised by CD45RA and FoxP3 (Fig. S1d), LC patients exhibited an increase in CD161^+^ Tregs (p < 0.0001; Fig. 1d), a marker preferentially expressed by FoxP3^lo^ Tregs (CD45RA^+^FoxP3^lo^ and CD45RA^−^FoxP3^lo^; Fig. S1e).^46^, ^47^, ^48^

We next investigated whether these phenotypic changes observed in LC Tregs, which are suggestive of lineage instability, were associated with functional abnormalities.^49^^,^^50^ First, we exposed HC and LC Tregs to pro-inflammatory cytokine mixtures. This resulted in a significant increase in the expression of IL-17, but not IFNγ by LC Tregs as compared to HCs (IL-17 Mix 1: p = 0.0055, Mix 2: p = 0.0015, IFNγ Mix 1: p = 0.044, Mix: 2 p > 0.99 Fig. 1e). Of note, at baseline there was a trend towards higher IL-17 and IFNγ expression by LC Tregs (IL-17 p = 0.11, IFNγ p = 0.28; Fig. S1f). Secondly, we determined the capacity of freshly isolated Tregs from LC and HC patients to suppress the proliferation of CFSE-labelled CD4^+^CD25^−^ third-party effector T cells. LC Tregs harboured a defective suppressive capacity as compared to HCs (Fig. 1f). Importantly, circulating Tregs isolated from patients with LC secondary to non-alcoholic steatohepatitis (NASH) with no prior alcohol history exhibited a degree of Treg functional deficit that was similar to Tregs from patients with alcohol-related LC (ARC), indicating that Treg impairment in LC is irrespective of the aetiology of liver disease (Fig. 1g). The magnitude of Treg dysfunction was inversely correlated with the severity of LC, as assessed by the MELD score (r = −0.82, p < 0.0001; Fig. 1h). Of note, no significant correlation was observed between Treg function and each individual constituent of the MELD score (serum creatinine, INR, bilirubin) (Fig. S1g). The phenotypic and functional abnormalities noted in the Tregs from LC patients were not associated with marked changes in the serum levels of circulating pro-inflammatory cytokines, which were found to be similar in HC and LC patients (Fig. S1h). In addition, the differences between LC and HC Tregs did not reflect changes in the epigenetic imprinting at the Treg-specific demethylated region (TSDR) of the FoxP3 locus (Fig. 1i).

### Circulating Tregs from LC patients exhibit increased intracellular ROS and changes in mitochondria morphology

Oxidative stress plays an important role in the pathophysiology of chronic liver diseases.^29^, ^30^, ^31^ Furthermore, experiments conducted in animal models indicate that oxidative stress can induce Treg apoptosis.^51^^,^^52^ Based on these studies, we sought to determine whether LC influences the redox homeostasis of Tregs, which may in turn provide an explanation for their increased apoptosis (Fig. 1c). As compared to HCs, Tregs from LC patients exhibited increased intracellular ROS levels (p = 0.0090; Fig. 2a), lipid peroxidation (p = 0.013; Fig. 2b), as well as a reduction in cell surface free thiol groups (p = 0.15; Fig. 2c), altogether supportive of a state of increased oxidative stress. We then investigated the two major sources of intracellular ROS, namely the membrane bound NADPH oxidases (Nox; particularly Nox2), and mitochondria, which in addition to producing ROS are also highly vulnerable to oxidative stress damage.^53^, ^54^, ^55^ We assessed the activation status of the pro-oxidant Nox2 by analysing the phosphorylation and co-localization of its subunits in the cell membrane.^56^ Confocal microscopy analysis of freshly isolated Tregs revealed that in LC the cytosolic factor p47^phox^ co-localized with the gp91^phox^ Nox2 subunit at the cell membrane considerably more than in HC Tregs, suggestive of a higher Nox2-associated ROS production (p = 0.049; Fig. 2d). In addition, levels of mitochondrial ROS were significantly higher in LC Tregs as compared to HC Tregs (p < 0.0001; Fig. 2e). To assess mitochondrial function of LC and HC Tregs, we performed metabolic flux analysis using the Seahorse Analyser (Fig. 2f). As compared to HC, LC Tregs had compromised mitochondrial respiration, with significantly reduced basal and maximal OCR (p = 0.028 and p = 0.058, respectively), as well as impaired ATP Production (p = 0.0008). These changes were paralleled by an increase in basal glycolysis (p = 0.12), decrease in glycolytic capacity (max. ECAR; p = 0.025) and a decrease in basal OCR/ECAR ratio (p = 0.0018; Fig. 2g). We also evaluated mitochondrial integrity by electron microscopy. As compared to HC, Tregs from LC patients displayed a significant increase in mitochondrial cristae width (p = 0.026; Fig. 2h), a well-established marker of impaired mitochondrial respiration and/or increased apoptosis.^57^, ^58^, ^59^

To further elucidate the molecular pathways underpinning these changes, we compared the transcriptional profile of freshly isolated HC and LC Tregs. Overall, the 2 cell populations differed in 267 differentially expressed genes (p < 0.01). Among the top 15 differentially regulated transcripts, we identified the gene encoding for the pro-apoptotic protein Bcl-2-modifying factor (*BMF*), a scaffolding protein involved in mitochondrial cristae integrity (*CHCHD3*), and the regulator of the mitochondrial redox sensor calcium-uniporter (*MCUB)* (Fig. 2i and Table S1). Gene set enrichment analysis (GSEA) revealed that LC Tregs exhibited an under-representation of gene sets involved in fatty acid metabolism, mitochondrial fatty acid beta oxidation and the pentose phosphate pathway (Fig. 2j and Table S2). These three pathways have been previously shown to be involved in Treg lineage stability, Treg function, and redox homeostasis, respectively.^60^, ^61^, ^62^, ^63^ Furthermore, LC Tregs showed enrichment in immune activation pathways such as IFNG, and under-representation of ribosome biogenesis pathways (Fig. 2k), which constitutes a hallmark of cell senescence and has been recently reported to be associated with decreased Treg proliferative capacity in vitro.^64^ Of note, Tregs expressing IFNγ and exhibiting reduced suppressive capacity are found in patients with autoimmune diseases such as multiple sclerosis.^65^

### The Nrf2/HO-1 redox pathway is dysregulated in Tregs from LC patients

The balance between ROS production and antioxidant signalling maintains a cell's redox homeostasis. In view of this, we next investigated whether alterations in the Nrf2 anti-oxidant signalling pathway could be an additional driver of the redox imbalance observed in LC Tregs. Whereas Nrf2 expression level was comparable in LC and HC Tregs (p = 0.59; Fig. 3a), Nrf2 nuclear translocation in response to Prostaglandin J2 (PGJ2) stimulation was significantly impaired in LC patients (HC p = 0.0009; LC p > 0.99; Fig. 3b). In line with this finding, LC Tregs exhibited substantially lower levels of HO-1 (p = 0.019; Fig. 3c and Fig. S2a), a trend towards higher levels of the HO-1 substrate Heme (Fig. S2b), and no significant changes in the protein expression of Bach1, which acts as a negative regulator of HO-1 (p = 0.71; Fig. 3d). To confirm the reduced Nrf2 activity, we quantified the transcript levels of two well-characterised targets of Nrf2, *NQO1* and *GCLM,* which were found to be lower in LC than in HC Tregs (*NQO1* p = 0.0012, *GCLM* p = 0.0028). *SOD1* did not differ between the two groups (p > 0.99), whereas no expression was detected for *GSTA2* and *GPX2* in our study cohort (Fig. 3e). The existence of a dysregulated Nrf2 pathway was further supported by the under-representation in the LC Treg transcriptome of the “Response to Oxygen radical” pathway, which contained key Nrf2 downstream targets among its core enrichment genes (*NQO1, CD36;* Fig. 3f).^14^^,^^66^

To determine the extent to which Nrf2 impacts on Treg phenotype and function, we evaluated the characteristics of Tregs isolated from mice with a germline deficiency of Nrf2 (Nrf2^−/−^). Tregs from Nrf2^−/−^ mice exhibited decreased protein expression of FoxP3 (p = 0.0042), CD39 (p = 0.033) and CTLA-4 (p = 0.039, Fig. 3g). Furthermore, in keeping with our observations in LC patients, Nrf2^−/−^ Tregs displayed an increased rate of apoptosis (p = 0.0007; Fig. 3h) and reduced suppressive function (Fig. 3i).

Having established the relevance of the Nrf2 pathway for Tregs, we investigated which of the downstream phenotypic and functional effects were directly caused by reduced HO-1. We used CRISPR/Cas9 to induce HO-1 deficiency in primary human Tregs and compared the results with what was observed in wild-type Tregs (WT) or when Tregs were electroporated with a CRISPR/Cas9 complex not targeting the human genome (NC). HO-1 knock-out efficiency was almost 80% (Fig. 3j), which was confirmed by assessing the proportion of inserts and deletions (Indels) induced by CRISPR/Cas9 in the *HMOX1* (HO-1) locus (Fig. S3). Knock-out of HO-1 did not influence the suppressive capacity of Tregs (Fig. 3k). In contrast, we observed that HO-1 was involved in Treg survival, given that, as compared to control cells, HO-1 knock-out Tregs were more prone to cell death when exposed to an environment of oxidative stress (Fig. 3l).

Taken together, these results indicate that an impaired Nrf2 pathway has a negative impact on the phenotype, function, and viability of Tregs, with the Nrf2 downstream target HO-1 being responsible for promoting Treg survival.

### Exposure to LC serum recapitulates some of the Treg functional abnormalities observed in LC patients

**Fig. 4:**
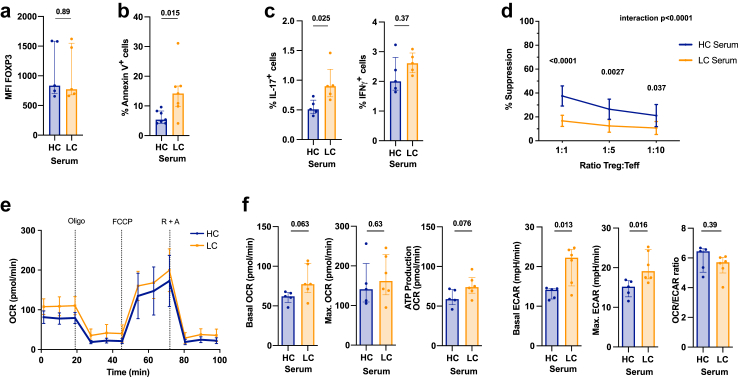
**Exposure to LC serum recapitulates some of the Treg functional abnormalities observed in LC patients. a.-e.** Healthy CD4^+^CD25^+^ Tregs were incubated with 25% HC or LC Serum for 24 h. **a.** FoxP3 expression (MFI). HC/LC serum n = 5. Unpaired t-test following log10 transformation. Median (IQR). **b.** Percentage of Annexin V^+^ Tregs. HC/LC serum n = 7. Unpaired t-test following log10 transformation. Median (IQR). **c.** Proportion of IL-17 (left) and IFNγ (right) expressing Tregs. HC/LC serum n = 5. Multiple unpaired t-tests with Bonferroni adjustment following log10 transformation. Median (IQR). **d.** Suppressive capacity of healthy Tregs after pre-incubating in 25% serum of HC or LC patients for 24 h. HC/LC serum n = 9. Two-way repeated measures ANOVA with Bonferroni's correction for multiple pairwise comparison. Values shown as SD. **e.** Metabolic profiling using Seahorse of CD4^+^CD25^+^ Tregs following incubation in 25% HC and LC Serum together with anti-CD3/CD28 beads (1:5 = bead to cell ratio) for 24 h. Oxygen Consumption Rate (OCR) and extracellular acidification rate (ECAR) was measured following the exposure to Oligomycin (Oligo), FCCP and Rotenone + Antimycin A (R + A). **f.** Left: basal and maximal OCR; and ATP production (measured after Oligomycin treatment) in HC and LC Tregs. Right: basal ECAR, glycolytic capacity (max. ECAR) and basal OCR/ECAR ratio in HC and LC Tregs. HC n = 9, LC n = 6. Unpaired t-tests following log10 transformation. Median (IQR). Abbreviations: Healthy control (HC), liver cirrhosis (LC), Oxygen consumption rate (OCR), Extracellular acidification rate (ECAR), Oligomycin (Oligo), Carbonyl cyanide-4 (trifluoromethoxy) phenylhydrazone (FCCP), Rotenone + Antimycin A (R + A).

Microenvironmental cues such as pro-inflammatory mediators or oxidative stress influence Treg function, survival, and phenotypic stability.^67^, ^68^, ^69^ We therefore sought to replicate the environment found in the circulation of LC patients by culturing healthy Tregs in LC patient serum for 24 h. Although culture in LC serum did not modify FoxP3 expression levels (p = 0.89, Fig. 4a), it did significantly increase the number of apoptotic Tregs (p = 0.015; Fig. 4b). In addition, LC serum induced a higher proportion of IL-17-, but not IFNγ-producing Tregs, which is indicative of Treg instability (IL-17 p = 0.025; IFNγ p = 0.37; Fig. 4c). The suppressive capacity of Tregs cultured with patient serum was reduced and mirrored the impaired function observed in LC Tregs (Fig. 4d). We also performed metabolic phenotyping of HC Tregs, using the Seahorse Analyser following a 24-h culture with LC or HC serum (Fig. 4e). As compared to HC serum, culture in the presence of LC sera increased basal glycolysis (p = 0.013) and the glycolytic capacity of HC Tregs (p = 0.016; Fig. 4e and f), with no significant changes in mitochondrial respiration being observed. Taken together, although culture of HC Tregs in LC serum for 24 h did not completely mirror the aberrant Treg phenotype and metabolic changes observed in LC, it recapitulated the reduced suppressive capacity of LC Tregs, which could be directly attributed to their enhanced glycolytic activity.^62^^,^^70^^,^^71^

## Discussion

Treg-orchestrated anti-inflammatory feedback loops are indispensable to maintain self-tolerance and immune homeostasis. Oxidative stress, via oxidation of membrane lipids and proteins, regulates cell death and contributes to the pathogenesis of all types of inflammatory disorders. The impact of redox balance on Tregs, however, has not been thoroughly elucidated and remains contentious. Thus, depending on the in vitro experimental conditions employed, Tregs have been shown to be either more resistant^72^ or conversely more vulnerable^67^ than other lymphocyte subsets to the cytotoxic effects of free oxygen species. We previously reported in mice that Nox2 deficiency enhances Treg suppression by promoting the nuclear translocation of FOXP3 and p65/NF-KappaB and increasing CTLA-4, CD39, CD73 and GITR protein levels.^12^ Given the relevance of activation of Nox2 as a source of ROS, these data provided an indirect proof of the harmful effects of oxidative stress on Treg phenotype and function. Our current study reveals that in patients with cirrhosis and liver failure, circulating Tregs exhibit increased oxidative mediated damage, evidenced by higher intracellular levels of ROS and lipid peroxidation with loss of mitochondrial integrity, which compromises their viability and functional properties.

Tregs rely on lipid metabolism and fatty acid oxidation for their survival and function.^61^^,^^73^ In this regard, a previous study identified oxidative-mediated mitochondrial damage as a mechanism of Treg dysfunction in patients with autoimmunity.^68^ Our data linking impaired redox homeostasis, mitochondrial damage, and compromised fatty acid beta oxidation, extend the observations originally made in autoimmunity to patients with LC. Furthermore, our findings provide a mechanistic explanation to account for these findings based on the imbalance between the two key non-mitochondrial regulators of intra-cellular redox homeostasis, namely the anti-oxidant Nrf2 pathway and the ROS-producing Nox2 complex. We previously described in cardiac cells that the activation of Nrf2 by NADPH oxidases is a key feed-back loop to prevent ROS build-up and oxidative-mediated cell damage.^74^ Additional experiments in glioneuronal cells showed that Nrf2 deficiency results in increased Nox2 activity and ROS levels.^75^ Our current findings indicate that in LC Tregs, the cross-talk between Nox2 and Nrf2 is severely disrupted due to reduced Nrf2 nuclear translocation upon stress. Of note, a dysregulated Nrf2 signalling can directly affect mitochondrial morphology and integrity.^76^^,^^77^ Furthermore, a defective Nrf2 system can disrupt both fatty acid oxidation^78^ and the pentose phosphate pathway.^63^ These published evidences, which are in keeping with the results of our electron microscopy and RNASeq experiments, respectively, further support the role of an impaired Nrf2 pathway as the key driver of the Treg abnormalities observed in LC.

In addition to the metabolic dysregulation and mitochondrial dysfunction outlined above, our study has identified an additional mechanism contributing to the reduced viability of Tregs in LC, which involves HO-1. The activity of cytoprotective enzyme HO-1 has been previously associated with Treg suppression^25^^,^^26^ and with the improvement in Treg function that results from prolonged cell culture in the presence of Rapamycin.^79^, ^80^, ^81^ By conducting gene deletion experiments we now demonstrate that HO-1 is a key determinant of human Treg survival when exposed to oxidative stress.

From the liver disease standpoint, our study reveals that an impaired Treg function is a yet unexplored feature of cirrhosis-associated immune dysfunction (CAID). To control for potential clinical confounders and unambiguously clarify the involvement of Tregs in CAID, we chose to study a well-defined cohort of cirrhotic patients at risk of clinical decompensations due to the presence of portal hypertension, but who were clinically stable at the time of analysis. The fact that the Treg deficit was detected even in patients who were clinically stable, had no signs of acute decompensation, and did not yet exhibit markers of systemic inflammation in the circulation, is an important aspect of our study that contrasts with most reports published to date on the pathogenesis of CAID, which have focused on very advanced forms of chronic liver disease (i.e. patients with acute decompensation and/or multi-organ failure).^82^ In view of the magnitude of the Treg functional deficit described here, and given the central role of this immune cell subset in preventing immune mediated disorders, we speculate that Treg dysfunction may predispose cirrhotic patients to develop systemic inflammation and immunopathology, by failing to counteract the effector function of key pro-inflammatory players such as cytotoxic T cells, monocytes and dendritic cells.

The Treg abnormalities described in our report are strikingly similar to what has been observed in paradigmatic autoimmune diseases such as type-1 diabetes, multiple sclerosis, and rheumatoid arthritis.^7^ A fundamental question that remains unanswered in autoimmunity is which are the mediators responsible for Treg dysfunction. Potential explanations include both cell intrinsic mechanisms linked to the patients' genetic susceptibility to autoimmunity and the effect of systemic inflammatory cues. In view of the result of culturing healthy Tregs with LC serum, cell extrinsic mediators influencing the Treg's redox balance machinery are much more likely to explain our findings than cell intrinsic mechanisms. The former could be related to the inflammatory microenvironment, metabolomic landscape, gut microbiome and/or the patients' nutritional status.

It is of importance to acknowledge that the study presented here has limitations derived from the use of relatively small sample sizes in some of the downstream experiments. As a result, our work cannot fully capture the clinical heterogeneity of the full spectrum of patients with chronic liver diseases. Future work conducted on larger patient cohorts will be required to account for therapy status, disease aetiology and stage and other confounders such as age and sex.

To conclude, by studying circulating Tregs from patients with stable cirrhosis we have uncovered a previously unrecognised immunoregulatory deficit that likely precedes the innate immune abnormalities that characterise CAID. Furthermore, we have gained insight into how the Nrf2-Nox2 signalling pathways and redox homeostasis influence Treg function and longevity. Our findings provide a strong rationale for exploring the therapeutic effects of Nrf2 activators and/or anti-oxidants in an attempt to modulate the inflammatory cascade at early stages of cirrhosis and potentially reduce the incidence of disease progression and clinical decompensations.

## Contributors

Study conceptualisation: NS, GL, ASF. Funding acquisition: NS. Data acquisition, analysis, and interpretation: EL, TV, NS, DMC, MR, SC, QP, EC, MC, JL, EPe, AZ, JE-H. Patient and Healthy control recruitment: TV, NS, LS, NSh. Reagent and material support: NL, RK. Verification of underlying data, figure preparation and writing of manuscript: NS, EL, TV, ASF. Critical revision of paper and important intellectual content: NS, EP, MT, MML, PN, AS, RIL, ASF, GL. All authors have read and approved the final version of the manuscript.

## Data sharing statement

Datasets are available from the corresponding author on reasonable request. The dataset generated by RNA-Sequencing is available on the Gene Expression Omnibus (GEO) public database (GEO identifier GSE234857).

## Declaration of interests

TV is currently employed by Janssen-Cilag Ltd (a subsidiary of Johnson & Johnson) and owns Johnson & Johnson stock/stock options. GL, ASF and MML are Founders of Quell Therapeutics Ltd. MR and MML are employed by Quell Therapeutics Ltd. PN received funding from Novo Nordisk; is advisory board member and received consulting fees from Novo Nordisk, Boehringer Ingelheim, Gilead, Intercept, Poxel Pharmaceuticals, BMS, Pfizer, Sun Pharma, Madrigal, GSK; speakers fees from Novo Nordisk, AiCME; and travel support from Novo Nordisk. The authors declare that the research in this manuscript was conducted in the absence of any commercial or financial relationships that could be construed as a potential conflict of interest.

## Appendix A Supplementary data

Supplementary Material Supplementary Figure 1. **Phenotypic characterisation of LC Tregs and correlation of their suppressive function with the constituents of the MELD score**. **a.** Representative flow cytometry plots indicating gating strategy of HC and LC PBMCs. **b.** Percentage of CD3^+^CD4^+^ T cells in HC and LC PBMCs. HC n = 7, LC n = 6; unpaired t test. Mean (SD). **c.** Percentage of CD4^+^CD25^+^ Tregs in HC and LC PBMCs expressing different phenotypic markers. n = 4. Multiple unpaired t-tests with Bonferroni adjustment following log10 transformation. Median (IQR). **d.** Proportion of FoxP3^+^ Tregs categorised as Population I (CD45RA^+^FoxP3^lo^), II (CD4RA^-^FoxP3^hi^), III (CD45RA^-^FoxP3^lo^). n = 10; Multiple unpaired t-tests with Bonferroni adjustment following log10 transformation. Median (IQR). **e.** Proportion of CD161^+^ cells within Population I, II and III of HC and LC Tregs. n = 10; Multiple unpaired t-tests with Bonferroni adjustment following log10 transformation. Median (IQR). **f.** Representative flowcytometry plot (left) and summary (%, right) of IL-17 and IFNγ expression in freshly isolated HC and LC Tregs. Multiple unpaired t-tests with Bonferroni adjustment following log10 transformation. Median (IQR). **g.** Linear regression of Treg suppression (from Fig. 1f) with Bilirubin, INR, and Creatinine, respectively, in LC patients. n = 9. Pearson’s correlation coefficient. **h.** Serum cytokine profile of HC and LC patients. HC n = 5, LC n = 9-10, unpaired t-tests with Welch’s correction where appropriate and Bonferroni adjustment for multiple comparisons following log10 transformation. No statistical test was applied to compare IL-12p40, IL-13 and IL-17A between HC and LC patients because less than 30% of the values were detectable in either of the group. Median (IQR). Abbreviations: Healthy control (HC), liver cirrhosis (LC), peripheral blood mononuclear cells (PBMCs). Supplementary Figure 2. **LC Tregs exhibit a dysregulated Nrf2 signalling evidenced by lower levels of HO-1 and resultant increased intracellular Heme**. **a.** HO-1 expression in freshly isolated Tregs of HC and LC measured by ELISA. HC n= 5, LC n= 6; unpaired t test with Welch’s correction following log10 transformation. Median (IQR). **b.** Heme concentration from cell lysates of freshly isolated Tregs from peripheral blood of HC and LC patients. HC n= 3, LC n= 4; unpaired t test following log10 transformation. Median (IQR). Abbreviations: Heme oxygenase-1 (HO-1), Healthy control (HC), liver cirrhosis (LC). Supplementary Figure 3. **Insertions and deletions at *HMOX-1* locus following CRISPR/Cas9-editing**. Percentage of insertions and deletions in the *HMOX1* (HO-1) locus at the cutting side of the CRISPR/Cas9 complex calculated by Tracking of Indels by Decomposition (TIDE) in HO-1 KO Tregs. n = 5.

Supplemental Western Blot
