# Supplementary material for: Dysregulated anti-oxidant signalling and compromised mitochondrial integrity negatively influence regulatory T cell function and viability in liver disease
Source: eBioMedicine. 2023 Aug 30;95:104778. doi: 10.1016/j.ebiom.2023.104778 (PMC10480539; doi:10.1016/j.ebiom.2023.104778)
Supplement: Supplementary Material — Supplementary Figure 1. Phenotypic characterisation of LC Tregs and correlation of their suppressive function with the constituents of the MELD score. a. Representative flow cytometry plots indicating gating strategy of HC and LC PBMCs. b. Percentage of CD3+CD4+ T cells in HC and LC PBMCs. HC n = 7, LC n = 6; unpaired t test. Mean (SD). c. Percentage of CD4+CD25+ Tregs in HC and LC PBMCs expressing different phenotypic markers. n = 4. Multiple unpaired t-tests with Bonferroni adjustment following log10 transformation. Median (IQR). d. Proportion of FoxP3+ Tregs categorised as Population I (CD45RA+FoxP3lo), II (CD4RA-FoxP3hi), III (CD45RA-FoxP3lo). n = 10; Multiple unpaired t-tests with Bonferroni adjustment following log10 transformation. Median (IQR). e. Proportion of CD161+ cells within Population I, II and III of HC and LC Tregs. n = 10; Multiple unpaired t-tests with Bonferroni adjustment following log10 transformation. Median (IQR). f. Representative flowcytometry plot (left) and summary (%, right) of IL-17 and IFNγ expression in freshly isolated HC and LC Tregs. Multiple unpaired t-tests with Bonferroni adjustment following log10 transformation. Median (IQR). g. Linear regression of Treg suppression (from Fig. 1f) with Bilirubin, INR, and Creatinine, respectively, in LC patients. n = 9. Pearson’s correlation coefficient. h. Serum cytokine profile of HC and LC patients. HC n = 5, LC n = 9-10, unpaired t-tests with Welch’s correction where appropriate and Bonferroni adjustment for multiple comparisons following log10 transformation. No statistical test was applied to compare IL-12p40, IL-13 and IL-17A between HC and LC patients because less than 30% of the values were detectable in either of the group. Median (IQR). Abbreviations: Healthy control (HC), liver cirrhosis (LC), peripheral blood mononuclear cells (PBMCs). Supplementary Figure 2. LC Tregs exhibit a dysregulated Nrf2 signalling evidenced by lower levels of HO-1 and resultant increased intracellular Hem [file mmc1.pdf]

Supplementary Material

Table of Contents

Supplementary Figure 1……………………………………………………………….2

Supplementary Figure 2……………………………………………………………….3

Supplementary Figure 3……………………………………………………………….3

Supplementary Table 1. Top differentially regulated genes in LC Tregs ………..….4

Supplementary Table 2. Transcriptional features of LC Tregs….……………………....5

Supplementary Table 3. Per-figure summary of applied statistical tests..…………..7

Supplementary Table 4. Summary of fluorochrome-conjugated antibodies………..11

Supplementary Figure 1.

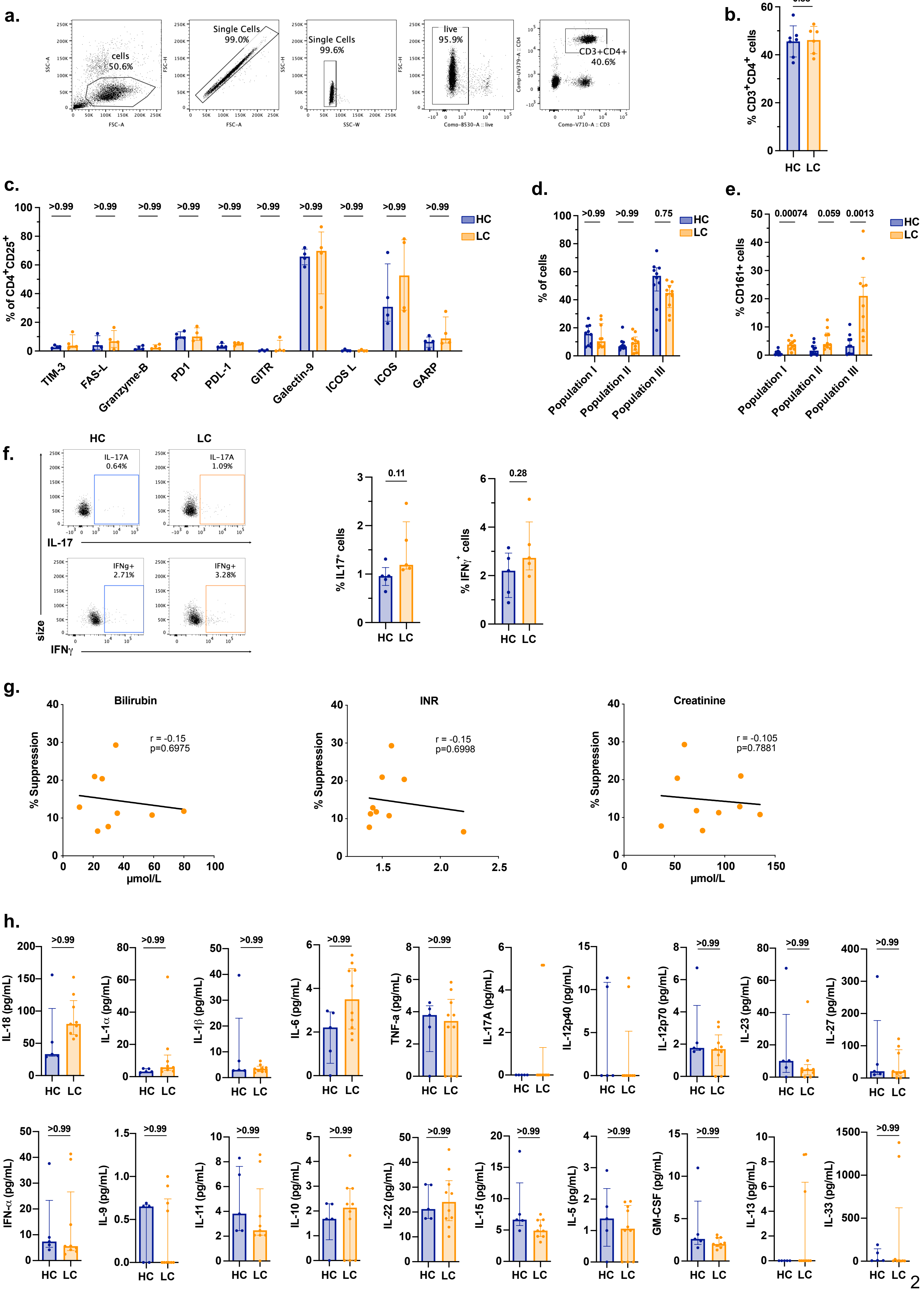

Supplementary Figure 2.

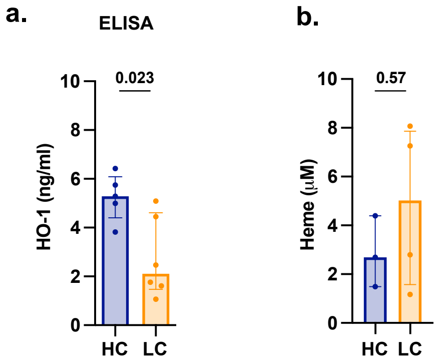

Supplementary Figure 3.

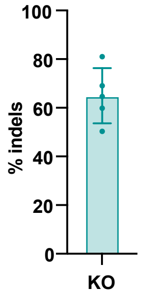

Supplementary Table 1.

Top 15 differentially expressed genes (p <0.01) between Tregs of liver cirrhotic patients (LC) and healthy controls (HC).

	Gene	Ensembl ID	log2r	p-value	q-value
**1**	*EBF4*	ENSG00000088881	-1.321376	0.000001	0.005725
**2**	*GPR15*	ENSG00000154165	2.100661	0.000001	0.005725
**3**	*HLA-H*	ENSG00000206341	2.095677	0.000003	0.022216
**4**	*LRRC37A3*	ENSG00000176809	1.429746	0.000005	0.023532
**5**	*SV2A*	ENSG00000159164	0.681124	0.000008	0.033876
**6**	*SPARC*	ENSG00000113140	-2.09316	0.000015	0.047453
**7**	*ARMC10*	ENSG00000170632	-0.87305	0.000017	0.047453
**8**	*CCND3*	ENSG00000112576	-0.680468	0.000019	0.047889
**9**	*PIK3C2B*	ENSG00000133056	0.840302	0.000026	0.057316
**10**	*MLLT3*	ENSG00000171843	-0.881823	0.000037	0.074275
**11**	*BMF*	ENSG00000104081	1.214842	0.000119	0.210555
**12**	*CHCHD3*	ENSG00000106554	-0.510803	0.000126	0.210555
**13**	*NRGN*	ENSG00000154146	-1.810251	0.000146	0.215613
**14**	*MCUB*	ENSG00000005059	-0.581298	0.000151	0.215613
**15**	*EDAR*	ENSG00000135960	-1.344782	0.000163	0.216792

Supplementary Table 2.

Top 15 downregulated canonical pathways (C2) in LC patients.

	ID	setSize	enrichmentScore	NES	pvalue	p.adjust	rank
1	KEGG_RIBOSOME	81	-0.581	-2.584	0.00004	0.0084	6231
2	REACTOME_EUKARYOTIC_TRANSLATION_ELONGATION	87	-0.571	-2.575	0.00004	0.0084	6231
3	REACTOME_EUKARYOTIC_TRANSLATION_INITIATION	111	-0.471	-2.215	0.00004	0.0084	5086
4	KEGG_PENTOSE_PHOSPHATE_PATHWAY	19	-0.654	-2.076	0.00036	0.0311	1851
5	REACTOME_ACTIVATION_OF_THE_MRNA_UPON_BINDING_OF_THE_CAP_BINDING_COMPLEX_AND_EIFS__AND_SUBSEQUENT_BINDING_TO_43S	55	-0.495	-2.043	0.00010	0.0124	5078
6	REACTOME_RESPONSE_OF_EIF2AK4_GCN2_TO_AMINO_ACID_DEFICIENCY	94	-0.446	-2.038	0.00004	0.0084	5086
7	REACTOME_SELENOAMINO_ACID_METABOLISM	109	-0.433	-2.028	0.00004	0.0084	5086
8	KEGG_PEROXISOME	70	-0.441	-1.908	0.00017	0.0187	4483
9	REACTOME_MISMATCH_REPAIR	15	-0.638	-1.898	0.00318	0.0997	2272
10	REACTOME_PURINE_CATABOLISM	17	-0.614	-1.892	0.00310	0.0997	1738
11	REACTOME_RESPONSE_TO_ELEVATED_PLATELET_CYTOSOLIC_CA2	107	-0.398	-1.860	0.00004	0.0084	1561
12	HALLMARK_FATTY_ACID_METABOLISM	134	-0.383	-1.855	0.00004	0.0084	2795
13	REACTOME_MITOCHONDRIAL_FATTY_ACID_BETA_OXIDATION	32	-0.506	-1.841	0.00170	0.0730	2539
14	REACTOME_METABOLISM_OF_NUCLEOTIDES	92	-0.396	-1.803	0.00030	0.0270	3165
15	REACTOME_NONSENSE_MEDIATED_DECAY_NMD	107	-0.364	-1.699	0.00043	0.0327	5086

Top 15 upregulated canonical pathways (C2) in LC patients.

	ID	setSize	enrichmentScore	NES	pvalue	p.adjust	rank
1	HALLMARK_INTERFERON_ALPHA_RESPONSE	96	0.605	2.462	0.00001	0.0084	4558
2	HALLMARK_INTERFERON_GAMMA_RESPONSE	193	0.508	2.272	0.00001	0.0084	2436
3	REACTOME_RIP_MEDIATED_NFKB_ACTIVATION_VIA_ZBP1	16	0.790	2.190	0.00003	0.0084	2018
4	REACTOME_ZBP1_DAI_MEDIATED_INDUCTION_OF_TYPE_I_IFNS	20	0.706	2.077	0.00005	0.0086	2018
5	REACTOME_RORA_ACTIVATES_GENE_EXPRESSION	17	0.679	1.914	0.00064	0.0422	4065
6	REACTOME_INTERFERON_ALPHA_BETA_SIGNALING	56	0.512	1.896	0.00006	0.0098	4546
7	REACTOME_TRANSPORT_OF_MATURE_TRANSCRIPT_TO_CYTOPLASM	79	0.474	1.869	0.00007	0.0102	4525
8	REACTOME_EXPORT_OF_VIRAL_RIBONUCLEOPROTEINS_FROM_NUCLEUS	33	0.559	1.856	0.00095	0.0535	4327
9	PID_CD40_PATHWAY	28	0.572	1.827	0.00147	0.0701	3475
10	REACTOME_DDX58_IFIH1_MEDIATED_INDUCTION_OF_INTERFERON_ALPHA_BETA	60	0.481	1.803	0.00042	0.0327	3551
11	REACTOME_NS1_MEDIATED_EFFECTS_ON_HOST_PATHWAYS	40	0.516	1.785	0.00174	0.0734	4327
12	REACTOME_INTERFERON_SIGNALING	178	0.402	1.780	0.00006	0.0099	4546
13	REACTOME_METABOLISM_OF_FAT_SOLUBLE_VITAMINS	35	0.529	1.779	0.00209	0.0795	1751
14	REACTOME_SUMOYLATION_OF_UBIQUITINYLATION_PROTEINS	39	0.516	1.776	0.00201	0.0795	5421
15	REACTOME_REGULATION_OF_GLUCOKINASE_BY_GLUCOKINASE_REGULATORY_PROTEIN	31	0.542	1.773	0.00247	0.0884	4327

Top 15 downregulated ontology gene sets (C5) in LC patients.

	ID	setSize	enrichmentScore	NES	pvalue	p.adjust	rank
1	GOBP_GLUCOSE_6_PHOSPHATE_METABOLIC_PROCESS	22	-0.662	-2.186	0.000	0.0245	3079
2	GOBP_RESPONSE_TO_OXYGEN_RADICAL	23	-0.639	-2.139	0.000	0.0245	1183
3	GOBP_CYTOPLASMIC_TRANSLATION	136	-0.439	-2.130	0.000	0.0234	5086
4	GOCC_INTRACILIARY_TRANSPORT_PARTICLE_B	18	-0.666	-2.086	0.000	0.0648	4341
5	GOCC_CYTOSOLIC_RIBOSOME	93	-0.456	-2.077	0.000	0.0234	5086
6	GOCC_T_CELL_RECEPTOR_COMPLEX	94	-0.449	-2.047	0.000	0.0234	4450
7	GOBP_PLATELET_AGGREGATION	61	-0.472	-1.983	0.000	0.0245	2724
8	GOCC_PLATELET_ALPHA_GRANULE_LUMEN	50	-0.491	-1.980	0.000	0.0379	1314
9	GOBP_CELLULAR_AMINO_ACID_CATABOLIC_PROCESS	94	-0.432	-1.971	0.000	0.0234	2871
10	GOBP_PLATELET_ACTIVATION	110	-0.415	-1.948	0.000	0.0234	2724
11	GOCC_CYTOSOLIC_SMALL_RIBOSOMAL_SUBUNIT	40	-0.506	-1.941	0.001	0.0835	5085
12	GOCC_PLATELET_ALPHA_GRANULE	70	-0.446	-1.927	0.000	0.0245	1314
13	GOBP_ALPHA_AMINO_ACID_CATABOLIC_PROCESS	78	-0.426	-1.880	0.000	0.0355	2871
14	GOCC_MICROBODY_LUMEN	45	-0.476	-1.876	0.001	0.0835	4408
15	GOBP_HEMOSTASIS	193	-0.353	-1.799	0.000	0.0239	2724

Top 15 upregulated ontology gene sets (C5) in LC patients.

	ID	setSize	enrichmentScore	NES	pvalue	p.adjust	rank
1	GOMF_HELICASE_ACTIVITY	149	0.463	2.006	0.000	0.0234	3554
2	GOBP_REGULATION_OF_VIRAL_GENOME_REPLICATION	81	0.499	1.975	0.000	0.0234	2353
3	GOBP_NEGATIVE_REGULATION_OF_VIRAL_GENOME_REPLICATION	53	0.535	1.959	0.000	0.0245	2353
4	GOBP_REGULATION_OF_RIG_I_SIGNALING_PATHWAY	19	0.664	1.928	0.001	0.0835	2291
5	GOMF_DNA_HELICASE_ACTIVITY	69	0.500	1.922	0.000	0.0234	2278
6	GOMF_ATP_DEPENDENT_ACTIVITY_ACTING_ON_RNA	75	0.490	1.910	0.000	0.0234	3554
7	GOBP_DEFENSE_RESPONSE_TO_SYMBIONT	246	0.405	1.858	0.000	0.0234	2602
8	GOCC_NUCLEAR_PORE	85	0.453	1.804	0.000	0.0427	4390
9	GOBP_DNA_GEOMETRIC_CHANGE	90	0.439	1.764	0.000	0.0598	2278
10	GOBP_RESPONSE_TO_VIRUS	330	0.366	1.725	0.000	0.0234	2606
11	GOBP_NUCLEAR_EXPORT	146	0.394	1.703	0.000	0.0450	3510
12	GOBP_HISTONE_METHYLATION	144	0.391	1.686	0.000	0.0652	3981
13	GOBP_MRNA_TRANSPORT	115	0.402	1.677	0.001	0.0985	4648
14	GOBP_PROTEIN_LOCALIZATION_TO_NUCLEUS	278	0.343	1.595	0.000	0.0257	6184
15	GOMF_HISTONE_BINDING	209	0.352	1.587	0.000	0.0756	3489

Supplementary Table 3. Per-figure summary of applied statistical tests.

Figure		*Mean (normal distribution) / Median (non-normal distriubtion)*	*SD (normal distribution) / IQR (non-normal distribution)*	*Data transformation (nominal or log10 transformed)*	*Statistical test applied*	*p-value*	*adjusted p-value*	*n-numbers HC*	*n-numbers LC*
**Figure 1**									
**1a**	% Tregs FACS			N/A	N/A	N/A		N/A	N/A
**1b**	% Tregs	Mean: HC 6.63%; LC 4.97%	SD: HC 1.18; LC 1.97	nominal	Unpaired t-test	0.0137		13	15
**1c**	% Annexin	Median: HC 0.39%; LC 8.53%	iQR: HC 2.94; LC 8.92	log10 transformed	unpaired t-test	0.0106		8	7
**1d**	Phenotype			log10 transformed	multiple unpaired t-tests and Bonferroni adjustment				
	FoxP3	Median: HC 92%; LC 88.2%	IQR: HC 3.65, LC 12.5			0.34	>0.99	17	15
	CD161	Median: HC 4.81; LC 14.8%	IQR: HC 3.44; LC 11.3			0.000006	0.00006	10	10
	CD62L CD27	Median: HC 79.75%; LC 71.45%	IQR: HC 11.13; LC 18.48			0.0056	0.056	10	10
	CD62L	Median: HC 89.65%; LC 86.9%	IQR: HC 5.65; LC 8.58			0.122	>0.99	10	10
	CD27	Median: HC 85.55%; LC 75.8%	IQR: HC 17.63; LC 14.9			0.053	0.53	10	10
	CXCR3	Median: HC 82.85%; LC 82.5%	IQR: HC 5.3; LC 17			0.55	>0.999	10	10
	CD39	Median: HC 42.6%; LC 27%	IQR: HC40.8; LC 47.1			0.062	0.62	17	19
	CTLA-4	Median: HC 21.6%; LC 10.7%	IQR: HC 10.43; LC 7.85			0.00097	0.0097	14	11
	Helios	Median: HC 64.3%; LC 46.3%	IQR: HC 23.2; LC 17.8			0.00022	0.0022	17	19
	HLA-DR	Median: HC 36.2%; LC 29.2%	IQR: HC 35; LC 29.35			0.39	>0.99	10	10
**1e**	IL17+ cells after cytokine mixes			log10 transformed	Two-way repeated measures ANOVA with Bonferroni's multiple comparison test			10	10
		Median: Mix 1. HC 1.55%; LC 5.39%	IQR: Mix 1. HC 1.72; LC 5.36		interaction p-value ANOVA (patient group HC/LC vs. Mixes)	0.71			
		Median: Mix 2. HC 0.99%, LC 3.78%	IQR: Mix 2. HC 2.53, LC 2.97		Mix 1		0.0055		
					Mix 2		0.0015		
	IFNg+ cells after cytokine mixes			log10 transformed	Two-way repeated measures ANOVA with Bonferroni's multiple comparison test			10	10
		Median: Mix 1. HC 3.7%; LC 6.015%	IQR: Mix 1. HC 1.97; LC 4.79		interaction p-value ANOVA (patient group HC/LC vs. Mixes)	0.083			
		Median: Mix 2. HC 4.78%, LC 4.64%	IQR: Mix 2. HC 3.72, LC 4.42		Mix 1		0.044		
					Mix 2		>0.99		
**1f**	Suppression			nominal	Two-way repeated measures ANOVA with Bonferroni's multiple comparison test			20	20
					interaction p-value ANOVA (patient group HC/LC vs. Treg:Teff ratio)	<0.0001			
	1:1	Mean: HC 20.17%; LC 71.29%	SD: HC 14.32; LC 10.54		1:1		<0.0001		
	1:5	Mean: HC 13.51; LC 40.59%	SD: HC 17.46; LC 15.45		1:5		<0.0001		
	1:10	Mean: HC 10.33%; LC 31.05%	SD: HC 14.24; LC 15.96		1:10		0.0003		
**1g**	Suppersion ACR vs. NASH			log10 transformed	Two-way repeated measures ANOVA with Bonferroni's multiple comparison test			5	5
					interaction p-value ANOVA (disease aetiology vs. Treg:Teff ratio)	0.7476			
	1:1	Mean: HC 27.68%; LC 14.75%<br>Median: HC 30.44% ; LC 11.89%	SD: HCC 12.72; LC 5.73;<br>IQR: HC 27.44; LC 10.66		1:1		0.3397		
	1:5	Mean: HC 4.07%; LC 6.31%<br>Median: HC 4.33%; LC 4.37%	SD: HC 3.29; LC 5.62;<br>IQR: HC 7.32; LC 9.41		1:5		>0.99		
	1:10	Median: HC 1.07%; LC 1.82%<br>Median: HC 0%; LC 2.68%	SD: HC 1.36; LC 3.04;<br>IQR: HC 2.68; LC 4.56		1:10		>0.99		
**1h**	Correlation MELD vs. Supp				Simple linear regression				20
					Pearson correlation coefficient r=-0.821				
**1i**	TSDR			N/A	N/A	N/A			
	CpG 44	Mean: Teff 94.98%; HC32.4%; LC 30.85%	SD: Teff 2.051; HC 21.49; LC 37.9						
	CpG 43	Mean: Teff 93%; HC 32.23%; LC 29.55%	SD: Teff 1.46; HC 20.9; LC 34.74						
	CpG 42	Mean: Teff 93.64%; HC 38.11%; LC 31.82%	SD: Teff 0.83; HC 25.57; LC 37.46						
	CpG 41	Mean: Teff 95.58%; HC 40.24%; LC 32.17%	SD: Teff 0.55; HC 26.77; LC 38.32						

	CpG 40	Mean: Teff 96.5%; HC 41%; LC 32.15%	SD: Teff 0.62; HC 25.79, LC 38.56						
	CpG 39	Mean: Teff 97.02%; HC 40.43%; LC 33.22%	SD: Teff 0.95; HC 25.38; LC 38.97						
	CpG 38	Mean: Teff 96.3%; HC 40.47%; LC 32.32%	SD: Teff 0.19; HC 26.44; LC 38.22						
	CpG 37	Mean: Teff 97.1%; HC 39.96%; LC 33.17%	SD: Teff 0.75; HC 24.92; LC 38.54						
	CpG 36	Mean: Teff 95.58%; HC 38.96%; LC 30.88%	SD: Teff 1.71; HC 25.27; LC 37.59						
	CpG 35	Mean: Teff 96.38%; HC 41.64%; LC 33.48%	SD: Teff 0.28; HC 26.63; LC 39.15						
	CpG 34	Mean: Teff 96.44%; HC 35.4%; LC 29.35%	SD: Teff 1.16; HC 24.04; LC 36.50						
**Figure 2**									
**2a**	MFI ROS	Median: HC 552.3; LC 1363	IQR: HC 369.9; LC 5781	log10 transformed	unpaired t-test with Welch's correction	0.009		8	7
**2b**	MFI Lipid Peroxidation	Median: HC 398.8; LC 790	IQR: HC 324.4; LC 6244	log10 transformed	unpaired t-test with Welch's correction	0.0134		8	7
**2c**	MFI Free Thiols	Median: HC 2034; LC 910	IQR: HC 2641.5; LC 839	log10 transformed	unpaired t-test	0.1484		8	7
**2d**	Colocalisation Nox2 subunits	Median: HC 0; LC 0.640	IQR: HC 0.1045; LC 0.7910	nominal	Mann-Whitney test	0.0492		5	7
**2e**	MFI MitoSox	Median: HC 29.35; LC 52.55	IQR: HC 13.92; LC 25.6	log10 transformed	unpaired t-test	<0.0001		12	16
**2f**	Seahorse							9	6
**2g**	Basal OCR	Median: HC 95.56; LC 44.34	IQR: HC 116.53; LC 39.85	log10 transformed	unpaired t-test	0.0281		9	6
	Max. OCR	Median: HC 329.1; LC 144.3	IQR: HC 467.7; LC 172.12	log10 transformed	unpaired t-test	0.0576		9	6
	ATP Production	Median: HC 75.39; LC 21.77	IQR: HC 115.04; LC 10.98	log10 transformed	unpaired t-test	0.0008		9	6
	Basal ECAR	Median: HC 68.71; LC 91.24	IQR: HC 33.91; LC 50.43	log10 transformed	unpaired t-test	0.1225		9	6
	Max. ECAR	Median: HC 17.99; LC 5.11	IQR: HC 27.42; LC 4.34	log10 transformed	unpaired t-test	0.025		9	6
	OCR/ECAR	Median: HC 1.93; LC 0.6108	IQR: HC 1.54; LC 0.2	log10 transformed	unpaired t-test	0.0018		9	6
**2h**	EM Mitochondria			log10 transformed	linear mixed effect model	0.026		3	3
**2i / 2j / 2k**	RNA Seq			N/A	N/A				
**Figure 3**									
**3a**	Nrf2WB	Mean: HC 0.58; LC 0.54	SD: HC 0.101; LC 0.14	nominal	unpaired t-test	0.5938		6	5
**3b**	Nrf2 nuclear translocation	-PGJ2 Mean: HC 0.5; LC 0.76	SD: HC 0.13; LC 0.14	nominal	Two-way repeated measures ANOVA with Bonferroni's multiple comparison test			5	5
		+PGJ2 Mean: HC 0.91; LC 0.73	SD: HC 0.29; LC 0.10		interaction p-value ANOVA (patient group HC/LC vs. PGJ2)	0.0027			
					-/+ PGJ2: HC		0.0009		
					-/+ PGJ2: LC		>0.99		
					HC vs LC (-PGJ2)		0.083		
**3c**	HO-1 WB	Median: HC 0.11; LC 0.0043	IQR: HC 0.16; LC 0.015	log10 transformed	unpaired t-test	0.0198		6	5
**3d**	Bach1 WB	Median: HC 0.66; LC 0.52	IQR: HC 0.78; LC 0.51	log10 transformed	unpaired t-test	0.714		7	5
**3e**	Nrf2 targets			log10 transformed	multiple unpaired t-tests with Bonferroni adjustment			15	12
	NQO1	Median: HC 1.57; LC 1.05	IQR: HC 0.33; LC 0.7			0.000393	0.001179		
	GCLM	Median: HC 1.37; LC 0.40	IQR: HC 1.41; LC 0.76			0.000949	0.002848		
	SOD1	Median: HC 0.75; LC 0.82	IQR: HC 0.29; LC 0.42			0.332385	0.997155		
**3f**	RNA Seq			N/A	N/A				
**3g**	Nrf2KO MFI FoxP3	Mean: HC 4520; LC 2955	SD: HC 297.8; LC 176.8	nominal	multiple unpaired t-tests with Bonferroni adjustment	0.0014	0.0042	3	3
	Nrf2KO MFI CD39	Mean: HC 1247; LC 908.3	SD: HC 128.5; LC 27.02	nominal	multiple unpaired t-tests with Bonferroni adjustment	0.0111	0.0333	3	3
	Nrf2KO MFI CTLA4	Mean: HC 972; LC 794.3	SD: HC 55.57; LC 45.94	nominal	multiple unpaired t-tests with Bonferroni adjustment	0.013	0.039	3	3
**3h**	Nrf2KO Annexin	Mean: HC 10.20%; LC 14.65%	SD: HC 1.73; LC 0.69	nominal	unpaired t-test	0.0007		5	5
**3i**	Nrf2KO Suppression	1:1 'Mean: HC 45.14%; LC 18.57%	SD: HC 4.22; LC 4.28	nominal	Two-way repeated measures ANOVA with Bonferroni's multiple comparison			7	7
		1:2 'Mean: HC 25.57%; LC 14.86%	SD: HC 3.69; LC 3.58		interaction p-value ANOVA (Treg Teff ratio vs. patient group HC/LC)	<0.0001			
					1:1	<0.0001			
					1:2	<0.0001			
**3j**	CRISPR KO efficiency	Median: WT 22.05%, NC 15.6%, KO 76.1%	IQR: WT 15.2, NC 29.2, KO 21.35	log10 transformed	ordinary one-way ANOVA with Bonferroni's multiple comparison test			WT 10, NC 11, KO 12	
					WT vs. NC		>0.99		
					WT vs. KO		0.0001		
					NC vs. KO		<0.0001		
**3k**	CRISPR suppression			nominal	Linear mixed effect model with Bonferroni's multiple comparison test			WT 4, NC 3, KO 4	
		1:1 Mean: WT 96.42%, NC 96.57%, KO 96.76 %	SD: WT 4.52, NC 4.35, KO 3.04		interaction p-value ANOVA (genotype vs. Treg Teff ratio)	0.7543			

		1:5 'Mean: WT 90.52%, NC 90.92%, KO 92.15%	SD: WT 7.85, NC 7.34, KO 5.71		1:1 - WT vs. NC		>0.99		
		1:10 'Mean: WT 79.54%, NC 78.74%, KO 86.34%	SD: WT 10.06, NC 11.27, KO 5.12		1:1 - WT vs. KO		>0.99		
		1:20 Mean: WT 45.7%, NC 57.79%, KO 57.05%	SD: WT 0.74, NC 24.06, KO 23.81		1:1 - NC vs. KO		>0.99		
					1:5 - WT vs. NC		>0.99		
					1:5 - WT vs. KO		>0.99		
					1:5 - NC vs. KO		>0.99		
					1:10 - WT vs. NC		>0.99		
					1:10 - WT vs. KO		0.8643		
					1:10 - NC vs. KO		>0.99		
					1:20 - WT vs. NC		>0.99		
					1:20 - WT vs. KO		>0.99		
					1:20 - NC vs. KO		>0.99		
**3l**	CRISPR viability			nominal	Two-way repeated measures ANOVA with Bonferroni's multiple comparison			WT 3, NC 3, KO 3	
		0uM: 'Mean: WT 58.57%, NC 55.17%, KO 53.7%	SD: WT 3.33, NC 1.76, KO 5.72		interaction p-value ANOVA (genotype vs. concentration)	0.054			
		40uM: 'Mean: WT 56.07%, NC 54.07%, KO 38.17%	SD: WT 1.35, NC 3.3, KO 9.53		0uM - WT vs. NC		>0.99		
		70uM: 'Mean: WT 52.17%, NC 50.5%, KO 32.27%	SD: WT 1.91, NC 1.7, KO 8.95		0uM - WT vs. KO		0.7819		
					0uM - NC vs. KO		>0.99		
					40uM - WT vs. NC		>0.99		
					40uM - WT vs. KO		0.0014		
					40uM - NC vs. KO		0.004		
					70uM - WT vs. NC		>0.99		
					70uM - WT vs. KO		0.0005		
					70uM - NC vs. KO		0.0012		
**Figure 4**									
**4a**	Serum: MFI FoxP3	Median: HC 836; LC 773	IQR: HC 882.5; LC 870	log10 transformed	unpaired t-test	0.8959		5	5
**4b**	Serum: AnnexinV	Median: HC 5.41%; LC 14.2%	IQR: HC 4.14; LC 6.83	log10 transformed	unpaired t-test	0.0151		7	7
**4c**	Serum: IL-17+ / IFNg	IL-17: Median: HC 0.51; LC 0.9	IQR: HC 0.23; LC 0.465	log10 transformed	multiple unpaired t-tests with Bonferroni adjustment			5	5
		IFNg: Median: HC 2.0; LC 2.61	IQR: HC 1.1; LC 0.665		IL-17	0.0123	0.0246		
					IFNg	0.1845	0.369		
**4d**	Serum: % Suppression			nominal	Two-way repeated measures ANOVA with Bonferroni multiple comparison test			9	9
					interaction p-value ANOVA (Treg Teff ratio vs. patient group HC/LC serum)	<0.0001			
		1:1 Mean: HC 37.57%; LC 16.75%	SD: HC 8.44; LC 4.74		1:1		<0.0001		
		1:5 Mean: HC 26.47%; LC 12.45%	SD: HC 8.43; LC 5.26		1:5		0.0027		
		1:10 Mean: HC 21.11%; LC 10.71%	SD: HC 9.27; LC 5.44		1:10		0.0374		
**4e**	Serum: Seahorse							5	6
	Basal OCR	Median: HC 60.90; LC 81.53	IQR: HC 12.67; LC 37.1	log10 transformed	unpaired t-test	0.0627			
	Max OCR	Median: HC 141; LC 162	IQR: HC 96.8; LC 102.1	log10 transformed	unpaired t-test	0.6277			
	ATP Production	Median: HC 58.91; LC 74.01	IQR: HC 19.28; LC 19.69	log10 transformed	unpaired t-test	0.0752			
	Basal ECAR	Median: HC 14.18; LC 22.25	IQR: HC 2.67; LC 9.19	log10 transformed	unpaired t-test	0.0131			
	Max ECAR	Median: HC 15.21; LC 19.14	IQR: HC 4.07; LC 7.36	log10 transformed	unpaired t-test	0.016			
	OCR/ECAR	Median: HC 6.45; LC 5.71	IQR: HC 1.581; LC 1.057	log10 transformed	unpaired t-test	0.3956			
**Supp. Fig. 1**									
**S1a**	Gating			N/A	N/A				
**S1b**	%CD3CD4	Mean: HC 45.60%; LC 46.13%	SD: HC 6.49; LC 5.69	nominal	unpaired t-test	0.88		7	6
**S1c**	Extended phenotype			log10 transformed	multiple unpaired t-tests with Bonferroni adjustment			4	4
	TIM-3	Median: HC 2.79%; LC 4.12%	IQR: HC 1.72; LC 8.89			0.294	>0.99		
	FAS-L	Median: HC 4.34%; LC 7.12%	IQR: HC 9.52; LC 11.84			0.553	>0.99		
	Granzyme B	Median: HC 2.14%; LC 2.5%	IQR: HC 2.56; LC 2.66			0.512	>0.99		
	PD1	Median: HC 10.5%; LC 10.43%	IQR: HC 4.78; LC 8.76			0.991	>0.99		
	PDL-1	Median: HC 3.1%; LC 5.49%	IQR: HC 2.48; LC 1.87			0.177	>0.99		
	GITR	Median: HC 0.395%; LC 0.54%	IQR: HC 0.785; LC 7.35			0.318	>0.99		
	Galectin-9	Median: HC 66.15%; LC 70.15%	IQR: HC 10.9; LC 43.12			0.719	>0.99		

	ICOS L	Median: HC 0.17%; LC 0.082%	IQR: HC 1.03; LC 0.84			0.380	>0.99		
	ICOS	Median: HC 31.15%; LC 52.95%	IQR: HC 40.08; LC 49.1			0.411	>0.99		
	GARP	Median: HC 6.735%; LC 9.12%	IQR: HC 7.16; LC 18.20			0.277	>0.99		
**S1d**	Miyara population			log10 transformed	multiple unpaired t-test with Bonferroni adjustment			10	10
		Population I: Median: HC 15.8%; LC 10.39%	IQR: HC 14.25; LC 16.43		Population I	0.63	>0.99		
		Population II: Median: HC 6.87%; LC 9.43%	IQR: HC 4.19; LC 9.39		Population II	0.958	>0.99		
		Population III: Median: HC 57.15%; LC 44.9%	IQR: HC 16.6; LC 15.9		Population III	0.251	0.754		
**S1e**	CD161 within Miyara populations			log10 transformed	multiple unpaired t-test with Bonferroni adjustment			10	10
		Populaton I: Median: HC 0.62%; LC 3.78%	IQR: HC 1.051; LC 2.88		Population I	0.000245	0.000736		
		Population II: Median: HC 1.61%; LC 3.94%	IQR: HC 3.16; LC 5.11		Population II	0.01998	0.0599		
		Population III: Median: HC 3.22%; LC 21%	IQR: HC 5.55; LC 19.32		Population III	0.000425	0.00127		
**S1f**	Baseline cytokine expression			log10 transformed	multiple unpaired t-test with Bonferroni adjustment			5	5
		IL-17: Median: HC 0.96 ; LC 1.19	IQR: HC 0.37 ; LC 0.975		IL-17+	0.0548	0.1096		
		IFNg: Median: HC 2.2 ; LC 2.73	IQR: HC 1.84 ; LC 1.98		IFNg+	0.1402	0.2804		
**S1g**	Correlation MELD components vs. Supp			nominal	Simple linear regression: Bilirubin	pearson coefficient r=0.1513			9
					Simple linear regression: INR	pearson coefficient r=0.1501			9
					Simple linear regression: Creatinine	pearson coefficient r=0.105			9
**S1h**	Cytokines in Serum								
	IL-18	Median: HC 33.27; LC 79.95	IQR: HC 74.1; LC 52.51	log10 transformed	unpaired t-test with Bonferroni adjustment for multiple comparison	0.053	>0.99	5	9
	IL-1 alpha	Median: HC 3.15; LC 5.770	IQR: HC 2.115; LC 9.54	log10 transformed	unpaired t-test with Welch's correction with Bonferroni adjustment for multiple comparison	0.0546	>0.99	5	9
	IL-1 beta	Median: HC 3.00; LC 3.43	IQR: HC 20.85; LC 2.13	log10 transformed	unpaired t-test with Welch's correction with Bonferroni adjustment for multiple comparison	0.3533	>0.99	5	9
	IL-6	Median: HC 2.21; LC 3.50	IQR: HC 2.36; LC 2.775	log10 transformed	unpaired t-test with Bonferroni adjustment for multiple comparison	0.1407	>0.99	5	10
	TNF-alpha	Median: HC 3.79; LC 3.425	IQR: HC 2.845; LC 4.773	log10 transformed	unpaired t-test with Bonferroni adjustment for multiple comparison	0.6919	>0.99	5	10
	IL-17A			N/A	N/A			5	10
	IL12p40			N/A	N/A			5	9
	IL12p70	Median: HC 1.76; LC 1.69	IQR: HC 2.795; LC 1.91	log10 transformed	unpaired t-test with Bonferroni adjustment for multiple comparison	0.6507	>0.99	5	9
	IL-23	Median: HC 10.06; LC 4.77	IQR: HC 35.93; LC 6.42	log10 transformed	unpaired t-test with Bonferroni adjustment for multiple comparison	0.2451	>0.99	5	9
	IL-27	Median: HC 20.96; LC 20.96	IQR: HC 166.94; LC 81.285	log10 transformed	unpaired t-test with Bonferroni adjustment for multiple comparison	0.8651	>0.99	5	9
	IFN-alpha	Median: HC 7.34; LC 5.55	IQR: HC 18.195; LC 22.72	log10 transformed	unpaired t-test with Bonferroni adjustment for multiple comparison	0.803	>0.99	5	9
	IL-9	Median: HC 0.65; LC 0	IQR: HC 0.67; LC 0.74	log10 transformed	unpaired t-test with Welch's correction with Bonferroni adjustment for multiple comparison	0.2613	>0.99	5	10
	IL-11	Median: HC 3.81; LC 2.47	IQR: HC 5.18; LC 3.74	log10 transformed	unpaired t-test with Bonferroni adjustment for multiple comparison	0.5041	>0.99	5	9
	IL-10	Median: HC 1.68; LC 2.13	IQR: HC 1.45; LC 2.913	log10 transformed	unpaired t-test with Bonferroni adjustment for multiple comparison	0.1794	>0.99	5	10
	IL-22	Median: HC 21.18; LC 24.05	IQR: HC 13.64; LC 16.11	log10 transformed	unpaired t-test with Bonferroni adjustment for multiple comparison	0.9808	>0.99	5	10
	IL-15	Median: HC 6.65; LC 4.89	IQR: HC 6.82; LC 2.64	log10 transformed	unpaired t-test with Bonferroni adjustment for multiple comparison	0.0591	>0.99	5	9
	IL-5	Median: HC 1.38; LC 1.060	IQR: HC 1.835; LC 1.798	log10 transformed	unpaired t-test with Bonferroni adjustment for multiple comparison	0.5608	>0.99	5	10
	GM-CSF	Median: HC 2.600; LC 1.990	IQR: HC 5.1; LC 1.06	log10 transformed	unpaired t-test with Welch's correction with Bonferroni adjustment for multiple comparison	0.2738	>0.99	5	9
	IL-13			N/A	N/A			5	10
	IL-33	Median: HC 8.870; LC 4.610	IQR: HC 137.3; LC 617.415	log10 transformed	unpaired t-test with Bonferroni adjustment for multiple comparison	0.7944	>0.99	5	9
**Supp. Fig.2**									
**S2a**	HO-1 ELISA	Median: HC 5.29; LC 2.11	IQR: HC 1.68; LC 3.136	log10 transformed	unpaired t-test with Welch's correction	0.0232		5	6
**S2b**	Heme	Median: HC 2.69; LC 5.025	IQR: HC 2.906; LC 6.286	log10 transformed	unpaired t-test	0.5737		3	4
**Supp. Fig.3**	Indels HO-1 KO cells			N/A	N/A				

Supplementary Table 4. Summary of fluorochrome-conjugated antibodies.

Target	*Antibody clone*	*Fluorochrome*	*Company*	*Cat. Nr.*	*RRID tag*
**Human:**					
CD3	OKT3	BV711	Biolegend	317328	AB_2562907
CD4	SK3	PercP	Biolegend	344624	AB_2563326
CD4	SK3	BUV395	BD Biosciences	563550	AB_2738273
CD4	RPA-T4	BV605	Biolegend	300556	AB_2564391
CD8	HIT8a	Pacific Blue	Biolegend	300928	AB_10612929
CD25	2A3	APC	BD Biosciences	340907	AB_2819021
CD25	CD25-4E3	PE	Thermo Fisher - eBioscience	12-0257-42	AB_2043825
CD25	M-A251	PE	BD Biosciences	555432	AB_395826
CD45RA	JS-83	FITC	Thermo Fisher - eBioscience	11-9979-42	AB_11151708
FOXP3	PCH101	FITC	Thermo Fisher - eBioscience	11-4776-42	AB_1724125
FOXP3	PCH101	PE	Thermo Fisher - eBioscience	12-4776-42	AB_1518782
FOXP3	236A/E7	PECF594	BD Biosciences	563955	AB_2738507
CD127	eBioRDR5	eFluor450	Thermo Fisher - eBioscience	48-1278-42	AB_1963566
CD127	A019D5	PECy7	Biolegend	351320	AB_10897098
CD127	A019D5	BV510	Biolegend	351332	AB_2562304
CCR6	R6H1	PeCy7	Thermo Fisher - eBioscience	25-1969-42	AB_1257138
CD161	HP-3G10	eFluor450	Thermo Fisher - eBioscience	48-1619-42	AB_10854273
CD27	O323	eFluor450	Thermo Fisher - eBioscience	48-0279-42	AB_10852844
CD62L	DREG-56	PeCy7	Biolegend	304822	AB_830801
HLA-DR	LN3	PeCy7	Thermo Fisher - eBioscience	25-9956-42	AB_1582284
CD39	eBioA1	PeCy7	Thermo Fisher - eBioscience	25-0399-42	AB_1582280
CD39	A1	APC/Cy7	Biolegend	328226	AB_2571981
ICOS-L	MIH12	PE	Thermo Fisher - eBioscience	12-5889-42	AB_10853668
CTLA-4	14D3	PE	Thermo Fisher - eBioscience	12-1529-42	AB_10805626
CTLA-4	BNI3	BV605	Biolegend	369610	AB_2632779
Granzyme-B	GB11	PE	Thermo Fisher - eBioscience	GRB04	AB_1500188
PD-1	eBioJ105	PE	Thermo Fisher - eBioscience	12-2799-42	AB_11042478
CD274 (PDL-1)	MIH-1	PeCy7	Thermo Fisher - eBioscience	25-5983-42	AB_1907368
GITR	eBioAITR	PE	Thermo Fisher - eBioscience	12-5875-42	AB_2572634
GARP	G14D9	eFluor660	Thermo Fisher - eBioscience	50-9882-42	AB_2574406
Helios	22F6	PE	Biolegend	137216	AB_10660749
Helios	22F6	PE/Cy7	Biolegend	137236	AB_2565990
CXCR3	G025H7	Pacific Blue	Biolegend	353724	AB_2561442
CD178 (FAS-L)	NOK-1	PE	Biolegend	306407	AB_2100664
TIM-3	F38-2E2	PE	Biolegend	345006	AB_2116576
HO-1	23	AF647	BD Biosciences	566391	AB_2739722
IL-17A	BL168	BV605	Biolegend	512326	AB_2563887
IFNg	4S.B3	PE/Cy7	Biolegend	502528	AB_2123323
**Murine:**					
CD3	145-2C11	APC	Thermo Fisher - eBioscience	17-0031-82	AB_469315
CD4	GK1.5	FITC	Thermo Fisher - eBioscience	11-0041-82	AB_464892
CD8	53-6.7,	eFluor450	Thermo Fisher - eBioscience	48-0081-82	AB_1272198
CD25	PC61.5,	eFluor780	Thermo Fisher - eBioscience	47-0251-82	AB_1272179
FoxP3	FJK-16s,	PE	Thermo Fisher - eBioscience	12-5773-82	AB_465936
CTLA-4	UC10-4B9	PECy7	Thermo Fisher - eBioscience	25-1522-82	AB_2688105
CD39	24DMS1	PercP-eFluor710	Thermo Fisher - eBioscience	46-0391-82	AB_10717953
