# Supplementary figures and images for: Dysregulated anti-oxidant signalling and compromised mitochondrial integrity negatively influence regulatory T cell function and viability in liver disease
Source: eBioMedicine. 2023 Aug 30;95:104778. doi: 10.1016/j.ebiom.2023.104778 (PMC10480539; doi:10.1016/j.ebiom.2023.104778)

# Supplemental Western Blot

## Nrf2

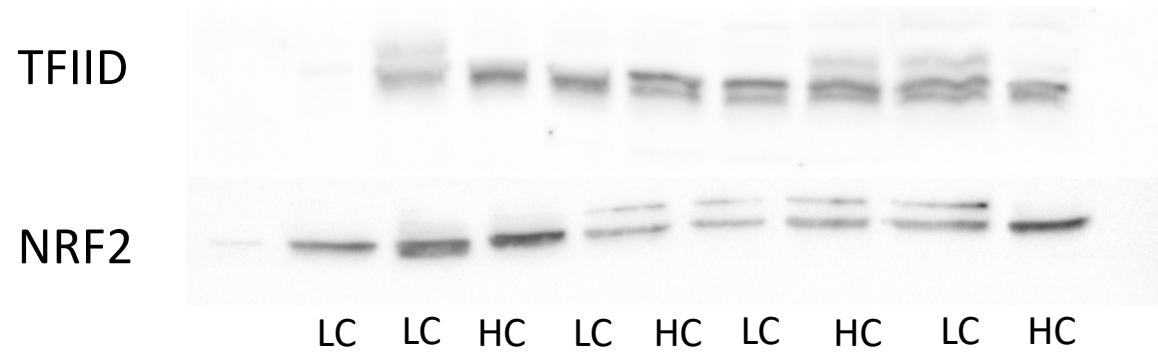

# HO-1

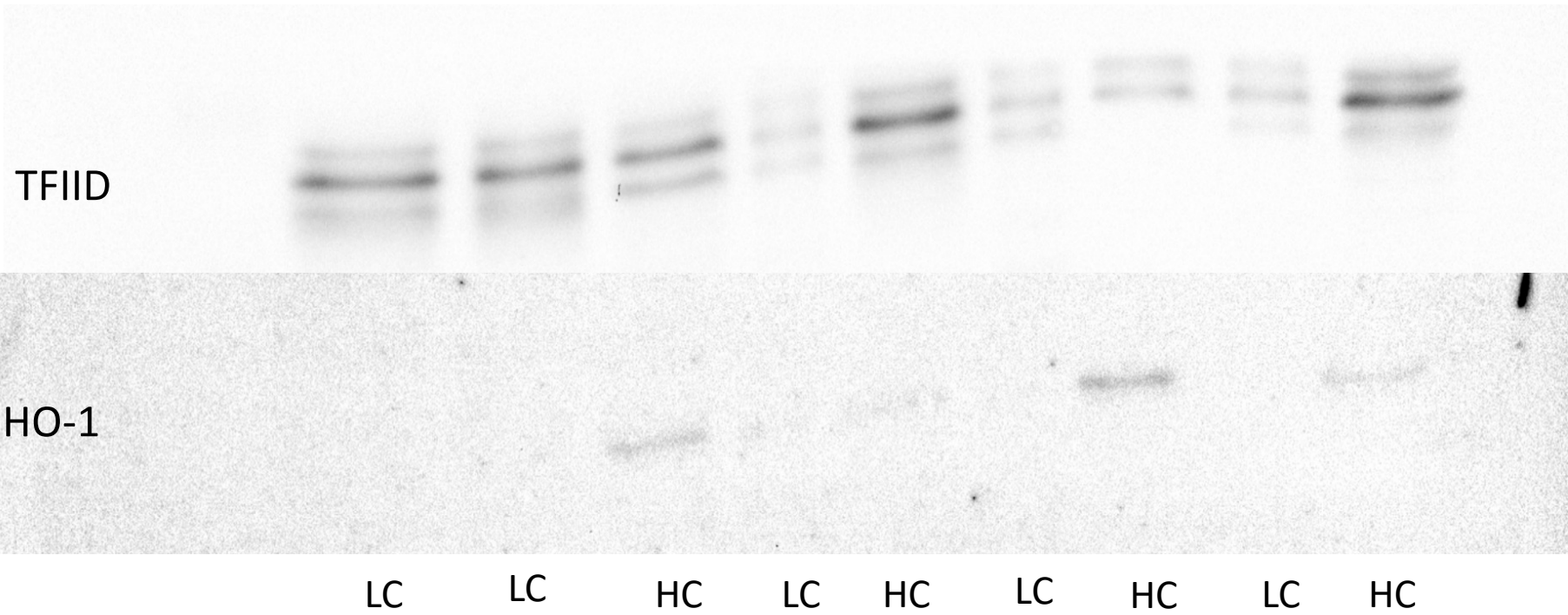

# Bach1

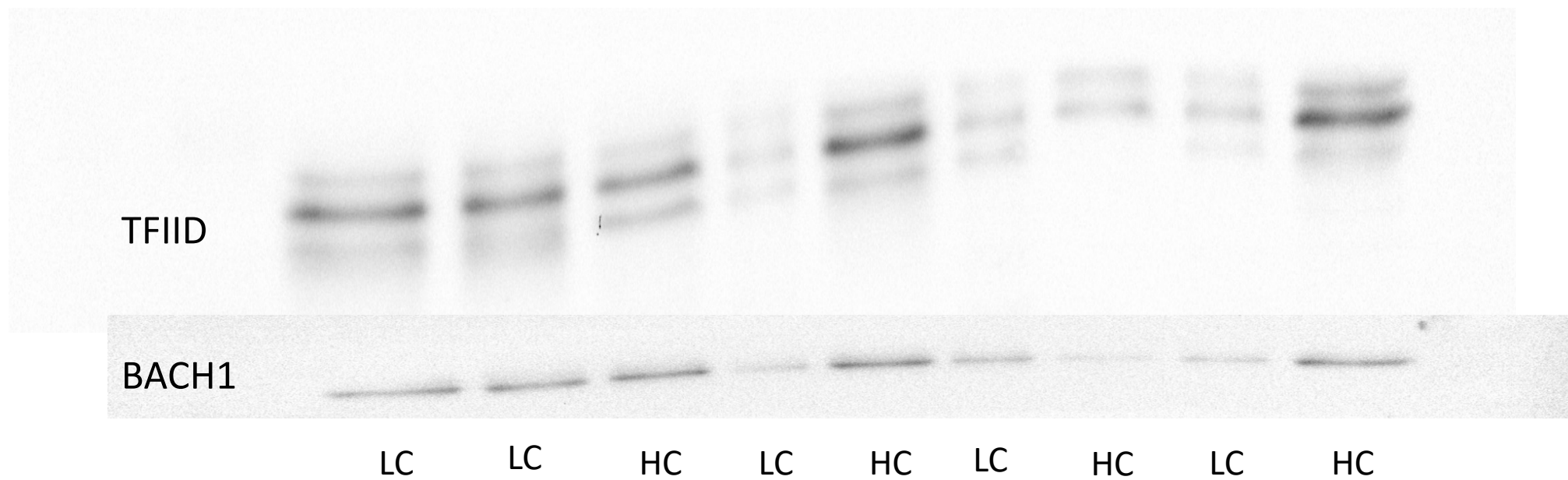

# Nrf2/HO-1/Bach1

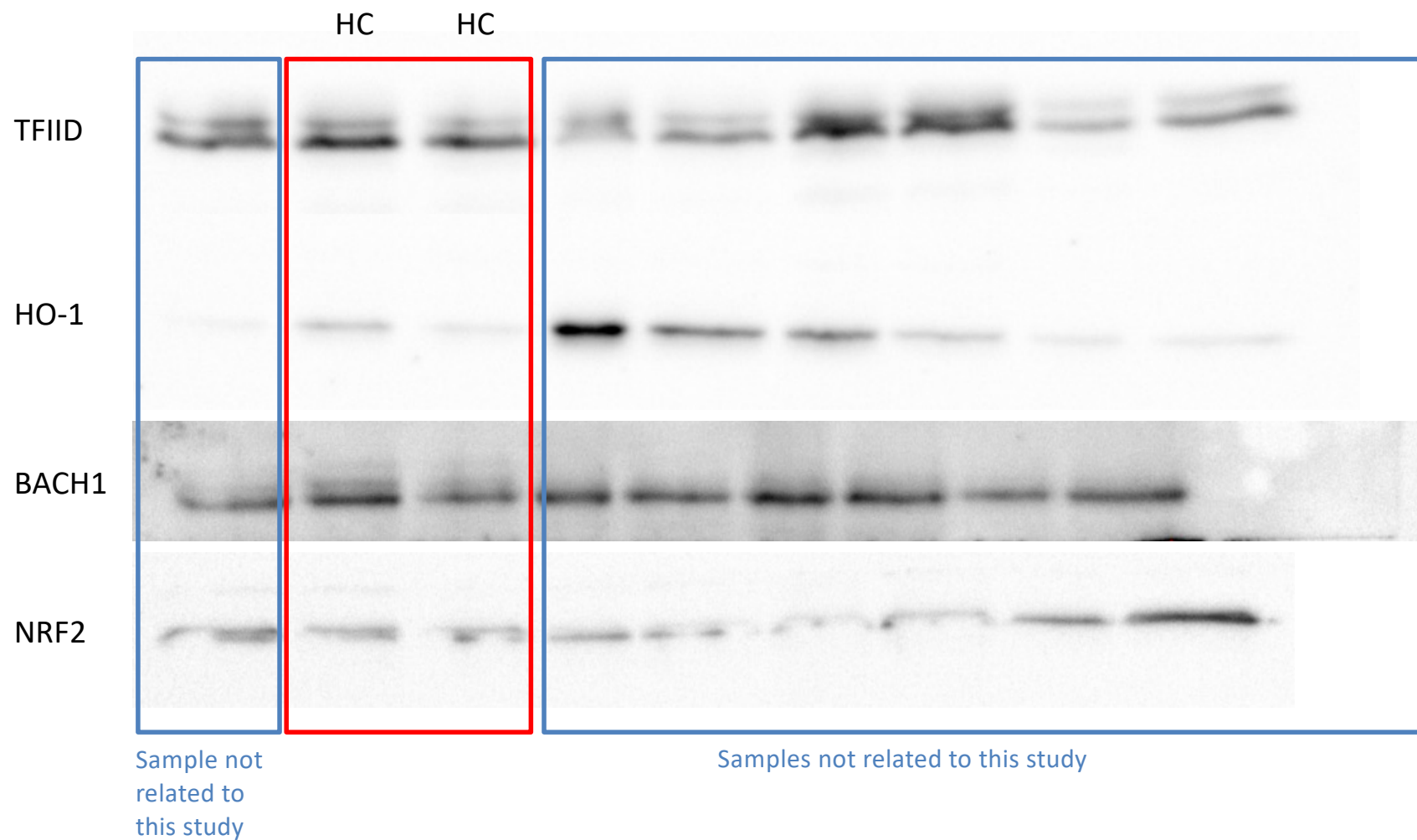

Supplement: Supplemental Western Blot [file mmc2.pdf]
